# Dynamics of Core Planar Polarity Protein Turnover and Stable Assembly into Discrete Membrane Subdomains

**DOI:** 10.1016/j.devcel.2011.03.018

**Published:** 2011-04-19

**Authors:** Helen Strutt, Samantha J. Warrington, David Strutt

**Affiliations:** 1 MRC Centre for Developmental and Biomedical Genetics, and Department of Biomedical Science, University of Sheffield, Western Bank, Sheffield S10 2TN, UK

## Abstract

The core planar polarity proteins localize asymmetrically to the adherens junctions of epithelial cells, where they have been hypothesized to assemble into intercellular complexes. Here, we show that the core proteins are preferentially distributed to discrete membrane subdomains (“puncta”), where they form asymmetric contacts between neighboring cells. Using an antibody internalization assay and fluorescence recovery after photobleaching in prepupal and pupal wings, we have investigated the turnover of two key core proteins, Flamingo and Frizzled, and find that the localization of both within puncta is highly stable. Furthermore, the transmembrane core proteins, Flamingo, Frizzled, and Strabismus, are necessary for stable localization of core proteins to junctions, whereas the cytoplasmic core proteins are required for their concentration into puncta. Thus, we define the distinct roles of specific core proteins in the formation of asymmetric contacts between cells, which is a key event in the generation of coordinated cellular asymmetry.

## Highlights

► Core polarity proteins form asymmetric cell-cell contacts at discrete membrane puncta ► Stable junctional localization of Fmi/Fz occurs without cytoplasmic core proteins ► Cytoplasmic core proteins promote concentration of Fmi/Fz into puncta ► Puncta are an important level of protein organization required for cellular asymmetry

## Introduction

Polarization of cells in a plane perpendicular to the apicobasal axis is a fundamental property of epithelia, and is of importance for multiple aspects of animal development. It is best characterized in the *Drosophila* wing, where each cell produces a single actin-rich trichome that points distally. A common group of “core planar polarity proteins” has been found to control planar polarity in *Drosophila* and other animals (Strutt, 2008; McNeill, 2010).

**Figure 1:**
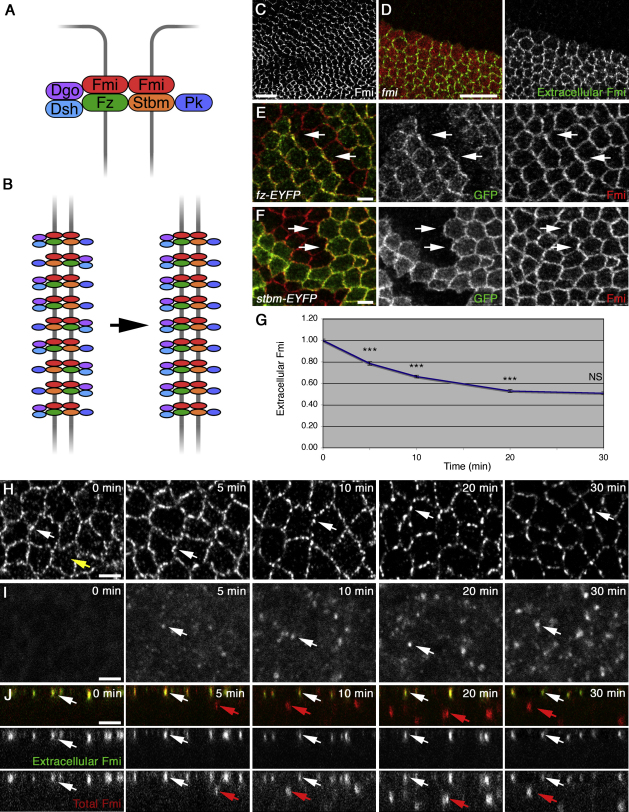
Asymmetric Localization of Core Proteins and Fmi Internalization in Prepupal Wings (A) Diagram of core protein distributions in inferred asymmetric intercellular complex. (B) Organization of individual complexes into domains of common polarity. (C–F) Prepupal wings. (C) Wild-type wing stained for Fmi. (D) *fmi^E59^* clone marked by loss of β-gal (red), stained for extracellular Fmi (green), showing that apical nonjunctional staining is specific. (E and F) *fz-EYFP* (E) and *stbm-EYFP* (F) mosaics, stained for GFP (green) and Fmi (red). Arrows show localization of Fz-EYFP toward (E) or Stbm-EYFP away from (F) the wing margin. (G–J) Fmi antibody internalization with chase times up to 30 min in wild-type prepupal wings. (G) Quantitation of extracellular Fmi staining. For each time point n > 100 in 19 experiments. Here and in later figures, error bars are standard error of the mean, and asterisks indicate p values (p^∗^ ≤ 0.05; p^∗∗^ ≤ 0.01; p^∗∗∗^ ≤ 0.001; NS, not significant p > 0.05). Here, p values are relative to the previous time point. (H) Extracellular Fmi staining in apical XY sections. Note that apical staining at 0 min (yellow arrow) is reduced at later times, whereas junctional puncta become more distinct (white arrows). (I) Total Fmi staining in subapical XY sections. Arrows indicate intracellular Fmi puncta. (J) XZ sections of extracellular (green) and total (red) Fmi staining, showing apical junctional puncta (white arrows) and intracellular Fmi puncta (red arrows). Scale bars, 10 μm (C and D) or 2.5 μm (E, F, and H–J). See also Figures S1 and S2.

In both flies and vertebrates, the core proteins localize asymmetrically within cells prior to morphological signs of polarization (Rida and Chen, 2009; Roszko et al., 2009; Strutt and Strutt, 2009; Hashimoto and Hamada, 2010). In the *Drosophila* early pupal wing, asymmetric localization is seen within the adherens junction region, oriented toward the wing margin (Classen et al., 2005; Aigouy et al., 2010). Cell rearrangements then lead to a remodeling of asymmetry, such that it aligns on the proximodistal (PD) axis (Aigouy et al., 2010). Consequently, at the time that trichomes emerge, the seven-pass transmembrane protein Frizzled (Fz) localizes distally, together with the cytoplasmic proteins Dishevelled (Dsh) and Diego (Dgo), whereas the transmembrane protein Strabismus (Stbm, also known as Van Gogh) and the cytoplasmic protein Prickle (Pk) localize proximally, and the seven-pass transmembrane cadherin Flamingo (Fmi, also known as Starry Night) localizes both proximally and distally (Figure 1A; reviewed in Strutt and Strutt, 2009).

A working model for the generation of this cellular asymmetry relies on both the establishment of molecular asymmetry, whereby the individual core proteins interact in an asymmetric complex spanning intercellular junctions, and the polarized distribution of such asymmetric complexes within cells (Figure 1B).

The view that the core proteins form an asymmetric complex is based principally on their patterns of localization. Aggregation experiments in S2 cells suggest that Fmi can interact homophilically via its extracellular cadherin repeats (Usui et al., 1999), and various other protein-protein interactions have been seen in vitro, or in transfected cells (reviewed in McNeill, 2010), but have proved difficult to demonstrate in vivo. Nevertheless, the notion of a complex is central to current models of planar polarity establishment (e.g., Amonlirdviman et al., 2005; Klein and Mlodzik, 2005; Le Garrec et al., 2006). Major questions remain regarding how asymmetric complexes are assembled and whether they are stable entities.

We previously showed that the transmembrane proteins are key to forming an intrinsically asymmetric complex in the pupal wing (Strutt and Strutt, 2008). In the absence of both Fz and Stbm, Fmi is not localized stably to junctions, and is instead found in the apical plasma membrane. However, Fz-Fmi in the junctions of one cell appear able to interact with Fmi in the neighboring cell, and the presence of both Fz and Stbm further increases Fmi localization to junctions. A plausible hypothesis is that in the absence of Fz and Stbm, Fmi is subjected to high rates of endocytic turnover, and that this turnover is inhibited once it forms asymmetric complexes with Fz and Stbm.

In support of this view, both Fmi and Fz appear subject to constant endocytic flux. Both are seen in Rab5-positive endocytic vesicles and accumulate when lysosomal degradation or recycling is blocked (Strutt and Strutt, 2008; Mottola et al., 2010). Furthermore, live imaging shows movement of large intracellular puncta containing Fz-EGFP across pupal wing cells (Shimada et al., 2006). The colocalization of these Fz-GFP puncta with the endosomal marker FM4-64 (Shimada et al., 2006), and experiments where Fmi-EGFP expression was transiently induced (Strutt and Strutt, 2008), both support the view that some of this vesicular Fmi and Fz is returning from the plasma membrane, rather than being newly synthesized. However, it remains to be established the degree to which plasma membrane populations of Fmi and Fz turn over in vivo, and how this is modulated by asymmetric complex formation.

How molecularly asymmetric complexes then become distributed in the same orientation at particular cell edges is also unknown: the most favored model is that it is a self-organizing process, dependent on feedback loops. Such a process would produce local cell-cell organization, which is thought to be globally organized by an upstream cue (reviewed in Klein and Mlodzik, 2005; Zallen, 2007; Strutt and Strutt, 2009; Vladar et al., 2009).

The cytoplasmic proteins Dsh, Pk, and Dgo are not necessary for localization of Fmi, Fz, and Stbm to junctions (Usui et al., 1999; Feiguin et al., 2001; Shimada et al., 2001; Strutt, 2001; Bastock et al., 2003), or for polarized signaling to neighboring cells (Strutt and Strutt, 2007; Chen et al., 2008). However, they are needed for the generation of cellular asymmetry through asymmetric localization of Fmi, Fz, and Stbm within the cell. We hypothesize that they may act chiefly by “clustering” asymmetric protein complexes of the same polarity.

To investigate further we have used the two independent approaches of antibody internalization assays and fluorescence recovery after photobleaching (FRAP) to analyze the turnover of the core proteins Fz and Fmi in pupal wings. We show that:

(1) Fz and Stbm cooperate to stably localize Fmi to junctions; and remaining “unstable” Fmi is removed by Rab5- and Dynamin-dependent processes.
(2) In the presence of the other core proteins, highly stable fractions of Fmi and Fz are concentrated in membrane subdomains (“puncta”). Cytoplasmic components such as Dsh and Pk are not required to generate the stable junctional fractions of Fz and Fmi but are necessary for their concentration into puncta, most likely by limiting lateral diffusion.
(3) Puncta are sites of asymmetric protein localization, and accumulation in puncta is correlated with the acquisition of cellular asymmetry.

Thus, we define the roles of the core proteins in a two-part model for establishment of planar polarity. First, Fz, Fmi, and Stbm can form intrinsically stable asymmetric complexes in the absence of the cytoplasmic components. Second, the cytoplasmic components promote “clustering” of asymmetric complexes into puncta, producing local domains of asymmetry.

## Results

### Measurement of Fmi Trafficking from the Cell Surface

To measure the gross endocytic turnover of Fmi, we used an antibody internalization assay on live pupal wing tissue. The core proteins are most strongly asymmetrically localized just before trichome initiation at around 28 hr after prepupa formation (APF) (Strutt and Strutt, 2009; Aigouy et al., 2010); however, at this stage the pupal wing is encased in cuticle that prevents antibody access. To circumvent this, prepupal wings (5–6 hr APF) were used. Asymmetric localization of the core proteins is seen at this stage (Figures 1C, 1E, and 1F; see also Classen et al., 2005; Aigouy et al., 2010), although it is less coherent than at 28 hr and oriented toward the wing margin. A monoclonal antibody against Fmi was used (Usui et al., 1999), which gives negligible background staining (Figure 1D).

Live prepupal wings were dissected in Schneider's Medium, and incubated at 4°C with antibody against Fmi, followed by washing and chasing at room temperature (RT) for up to 30 min before fixation. The amount of Fmi remaining at the cell surface was determined via incubation with secondary antibody in the absence of detergent, revealing a reduction over time (Figures 1G and 1H; and see Figure S1A available online). This loss of extracellular staining reflects internalization of Fmi because no antibody dissociated in control experiments in which tissue was fixed prior to antibody incubation (Figure S1B). Furthermore, incubation with secondary antibody in the presence of detergent revealed that the loss of extracellular Fmi was accompanied by the appearance of Fmi antibody in intracellular puncta, which were initially seen just below the apical junctions, and spread more basally over time (Figures 1I and 1J; Figure S2A–S2E).

Crosslinking of Fmi by the bivalent whole antibody could cause clustering that might either stimulate or inhibit internalization; therefore, we generated Fab antibody fragments. These Fab fragments stained live prepupal wings similarly to full-length antibody (Figure S1E); however, the binding affinity was poorer, and antibody dissociated over time (40% over 20 min, Figure S1B). Nevertheless, internalization was evident because the loss of extracellular staining was greater than could be accounted for by antibody falloff (Figures S1C and S1C′), and Fmi Fab antibody was detected in intracellular puncta (Figures S1F–S1H).

Extracellular Fmi staining decreased rapidly within the first 10 min, and subsequently the rate slowed (Figure 1G). This does not reflect a general defect in endocytosis due to prolonged culture because Dextran internalization was similar throughout the chase period (Figures S1I and S1J). Instead, this may be due to either recycling of internalized Fmi to the cell surface, or the presence of a stable population(s) of Fmi at the cell surface that is resistant to endocytosis.

### Internalized Fmi Enters Both Degradative and Recycling Pathways

**Figure 2:**
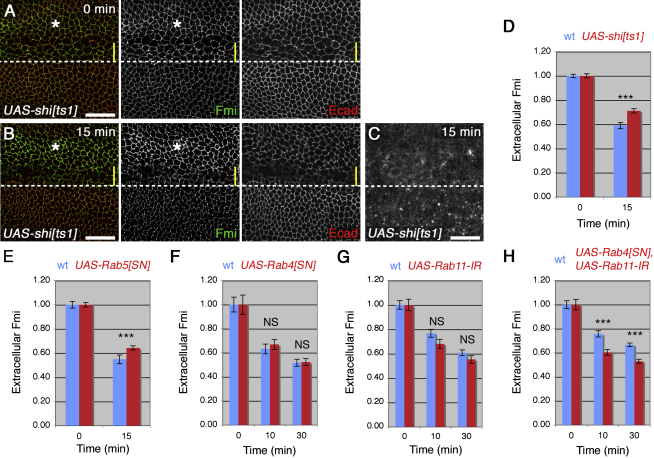
Trafficking of Internalized Fmi (A–C) *ptc-GAL4, UAS-shi^ts1^* prepupal wings, raised at 34°C for 2 hr before dissection, *ptc-GAL4* domain above the dotted line. (A and B) Extracellular Fmi staining (green) and E-cadherin staining (red) with 0-min (A) and 15-min (B) chase at 34°C. Quantitation was performed on the region marked by the asterisk, outside the most highly expressing region where E-cadherin staining was disrupted and, thus, not suitable for quantitation (yellow bar). (C) Subapical section, total Fmi staining with 15-min chase. Scale bars, 20 μm. (D–H) Quantitation of extracellular Fmi staining in prepupal wings, with UAS constructs expressed in the *ptc-GAL4* domain. Asterisks indicate p values of mutant relative to wild-type at the same time point. See also Figure S3.

To investigate further we tested whether Fmi internalization occurred via Dynamin-dependent endocytosis. Expression of a temperature-sensitive allele of *shibire* (*shi*, the *Drosophila* gene encoding Dynamin*)* at the restrictive temperature caused a subtle accumulation of extracellular Fmi (1.4-fold above wild-type tissue, Figures 2A; Figure S3C). The small increase in steady-state levels of Fmi at junctions may reflect feedback, in which reduced endocytosis is balanced by reduced delivery of Fmi to the plasma membrane. Nevertheless, after a 15-min chase to allow internalization of antibody-bound Fmi, a greater proportion of extracellular Fmi remained in *shi* mutant tissue than in wild-type tissue (30% less internalization), and also fewer intracellular puncta were seen (Figures 2B–2D; Figures S3A and S3B). The residual internalization of Fmi is likely to be in part due to incomplete loss of *shi* activity in the region quantified, although we cannot exclude the possibility of Dynamin-independent endocytosis of Fmi. We conclude that Fmi internalization is at least partially Dynamin dependent.

Expression of dominant-negative Rab5 (Rab5^SN^) for 2 hr also reduced Fmi internalization (20% less internalization, Figure 2E); again the incomplete blockage of Fmi endocytosis probably reflects the incomplete loss of Rab5 activity (Figure S3D). Consistent with Fmi being trafficked via Rab5, a subset of internalized Fmi colocalized strongly with Rab5 vesicles (Figure S3F). Internalized Fmi also partially colocalized with Rab7 (Figure S3G), and excess accumulation of internalized Fmi was seen when lysosomal maturation was disrupted in *deep orange* (*dor*) clones (Figure S3E), suggesting that Fmi enters a degradative pathway.

The slowing in the rate of Fmi loss from the cell surface over time could indicate that some Fmi is normally recycled to the plasma membrane, either via the fast Rab4-dependent pathway, or the slow Rab11-dependent mechanism. Expression of either dominant-negative Rab4 alone or Rab11 RNAi alone did not significantly affect Fmi internalization (Figures 2F and 2G). However, coexpression of Rab11 RNAi and dominant-negative Rab4 led to a more rapid loss of Fmi from the cell surface than in wild-type tissue (Figures 2H; Figure S3M–S3Q).

Only occasional colocalization was seen between internalized Fmi puncta and Rab11 (Figure S3H), and we do not have a reliable marker for the Rab4 compartment (see legend to Figure S3M). However, when dominant-negative Rab4 was expressed, more frequent (although still rare) colocalization of Fmi puncta with Rab11 was seen (Figure S3I). These data are consistent with Fmi normally being recycled via the rapid Rab4 pathway. Most likely, loss of Rab4 does not block Fmi recycling but shifts it into the slower Rab11 pathway.

### A Population of Fmi Localizes Persistently to Junctional Puncta

**Figure 3:**
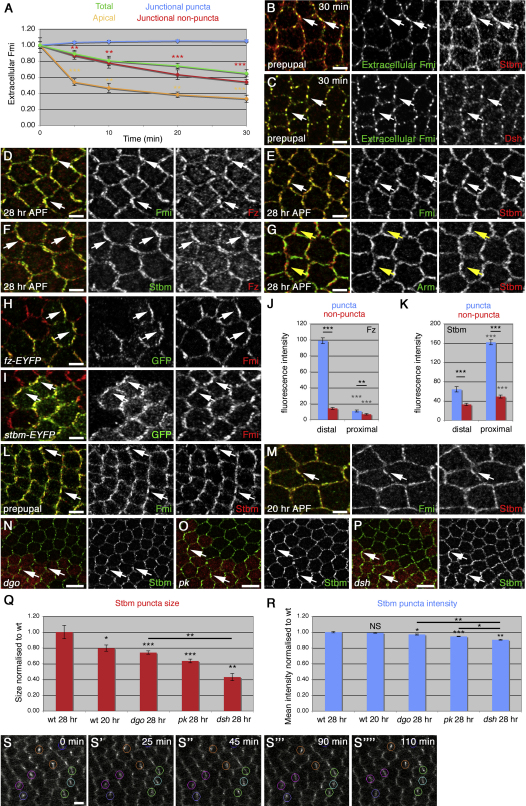
Asymmetric Localization of Core Proteins in Junctional Puncta (A) Fmi antibody internalization with chase times up to 30 min in wild-type wings. Quantitation of extracellular Fmi staining in junctional puncta, junctional nonpuncta, or the apical plasma membrane, or total (see Figure 1G). Asterisks indicate p values comparing Fmi levels between junctional puncta and nonpuncta (red) or between junctional nonpuncta and the apical membrane (orange). The mean intensity of Fmi staining in puncta does not change over time nor does the size or number of puncta (not shown). (B and C) Extracellular Fmi staining (green) after a 30-min chase in prepupal wings, colabeled with total Stbm (B) or Dsh (C) staining (red). Arrows point to prominent junctional puncta. (D–G) Twenty-eight hour pupal wings, stained for: (D) total Fmi (green), Fz (red); (E) total Fmi (green), Stbm (red); (F) Stbm (green), Fz (red); and (G) Arm (green), Stbm (red). White arrows indicate strongly colocalizing puncta; yellow arrows indicate Stbm puncta that do not colocalize with Arm. (H and I) *fz-EYFP* (H) and *stbm-EYFP* (I) mosaics in 28-hr wings, stained for GFP (green) and Fmi (red). Arrows indicate distal (H) or proximal (I) puncta. (J and K) Quantitation of fluorescence intensity of Fz-EYFP (J) or Stbm-EYFP (K) staining in proximal and distal puncta and nonpuncta in mosaics. Puncta were selected on the basis of Fmi staining. Asterisks are p values comparing proximal and distal puncta or nonpuncta (gray), or as indicated by bars. Weak Fz and Stbm PD asymmetry is seen in nonpuncta (2.0- and 1.5-fold, respectively), but asymmetry is stronger within puncta (9.0- and 2.5-fold, respectively). (L and M) Prepupal (L) or 20-hr (M) pupal wings stained for Fmi (green) and Stbm (red), showing typical puncta (arrows). (N–P) Twenty-eight hour pupal wings containing clones of *dgo^380^* (N), *pk^pk-sple13^* (O), or *dsh^V26^* (P), marked by loss of β-gal (red), stained for Stbm (green). Arrows point to puncta in wild-type tissue. Puncta are less prominent in mutant tissue. (Q and R) Quantitation of puncta size (Q) or mean fluorescence intensity (R) in 28- and 20-hr wild-type pupal wings, and in *dgo^380^*, *pk^pk-sple13^*, and *dsh^V26^* mutant clones stained for Stbm. Asterisks are p values compared to 28-hr wild-type wings, or as indicated by bars. (S–S″″) Time-lapse images of 27- to 29-hr pupal wing, imaged over a 2-hr period. Puncta (representative examples circled) persist over time. Scale bars, 2.5 μm (B–I, L, and M) or 5 μm (N–P and S). See also Figure S4 and Movie S1.

Although our data show that recycling contributes to the observed slowing of removal of cell surface Fmi, we also find evidence for a population of Fmi that is resistant to endocytosis. At 0 min extracellular Fmi localized both at apical junctions, where it is enriched in punctate structures, and also to the apical plasma membrane (Figure 1H). However, by 30 min, the only detectable extracellular Fmi staining was in discrete puncta within the apical junctions (Figure 1H; Figures S1E and S2A–S2E). This suggests that in addition to apical and junctional nonpuncta populations of Fmi that are subject to endocytosis, there is a junctional puncta population of Fmi subject to little or no endocytosis. Quantitation of the different Fmi populations revealed that there was no change in the mean intensity of Fmi staining in junctional puncta, whereas Fmi from junctional regions lacking puncta was internalized at an intermediate rate, and apical Fmi was rapidly internalized (Figure 3A). Thus, there is a persistent plasma membrane fraction of Fmi concentrated in junctional puncta. Notably, this colocalizes strongly with other core proteins (Figures 3B and 3C; data not shown).

High-resolution images of 28-hr pupal wings showed that at this stage, proximally and distally localizing polarity proteins were also concentrated in puncta in PD junctions (Figures 3D–3F; see also Aigouy et al., 2010). Furthermore, mosaic analysis showed that puncta are the major sites of asymmetry (Figures 3H–3K; Figure S4A). Interestingly, these puncta define a distinct membrane compartment that does not colocalize with other adherens junction markers such as Armadillo and E-cadherin (Figure 3G; data not shown).

**Figure 4:**
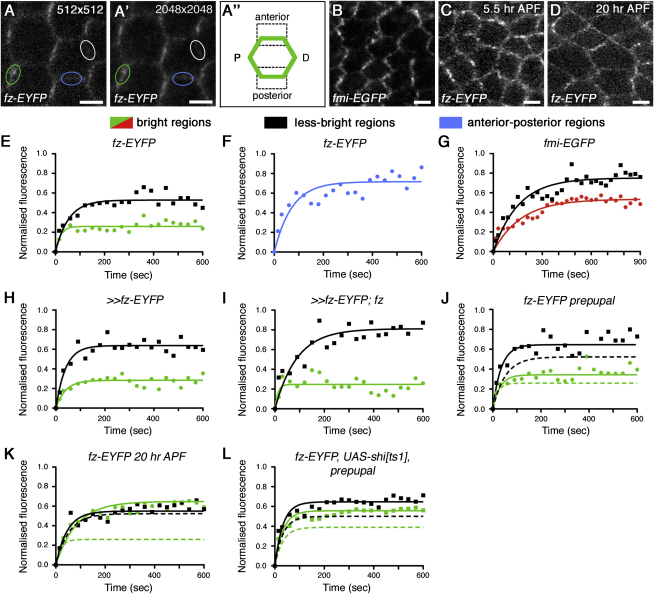
FRAP Analysis of Junctional Fz-EYFP and Fmi-EGFP (A) Images showing a 28-hr live wing expressing *ActP-fz-EYFP/+* at the resolution used in FRAP experiments (pixel size 139 nm) (A) or higher resolution (pixel size 35 nm) (A′). Ovals indicate a typical bright region containing predominantly puncta (green), a less-bright region containing mostly nonpuncta (white), or an anterior-posterior (AP) boundary where no puncta are seen (blue). Scale bars, 5 μm. (A″) Cartoon of a pupal wing cell. (B–D) Live wings expressing *ArmP-fmi-EGFP/+* at 28 hr APF (B) or *ActP-fz-EYFP/+* in prepupal wings (C) and at 20 hr APF (D). Scale bars, 2.5 μm. (E–L) FRAP analysis on PD boundary bright regions (green, red), PD boundary less-bright regions (black), or AP boundaries (blue) of junctions in prepupal wings (J and L), 20-hr wings (K), or 28-hr wings (E, F, H, and I) expressing Fz-EYFP, or 28-hr wings expressing Fmi-EGFP (G). (E and F) *ActP-fz-EYFP/+*, PD-localized (E) or AP localized (F). (G) ArmP-fmi-EGFP/+. (H and I) *Ubx-FLP; ActP-FRT-polyA-FRT-fz-EYFP/+* (H) *or Ubx-FLP; ActP-FRT-polyA-FRT-fz-EYFP/+; fz^P21^* (I). Less-bright regions have a significantly smaller stable fraction when activation of *fz-EYFP* expression is delayed or *fz* dosage is reduced, p^∗∗∗^ for *ActP-fz-EYFP/+* (E) compared to *Ubx-FLP; ActP-FRT-polyA-FRT-fz-EYFP/+* (H), p^∗∗∗^ for *ActP-fz-EYFP/+* (E) compared to *Ubx-FLP; ActP-FRT-polyA-FRT-fz-EYFP/+; fz^P21^* (I), p^∗^ for *Ubx-FLP; ActP-FRT-polyA-FRT-fz-EYFP/+* (H) compared to *Ubx-FLP; ActP-FRT-polyA-FRT-fz-EYFP/+; fz^P21^* (I). (J and K) *ActP-fz-EYFP/+* in prepupal (J) and 20 hr APF (K) wings. Dotted lines indicate 28-hr wing data, for comparison (see E). The stable fraction of bright regions is significantly different at each time point: p(prepupal–28 hr)^∗∗∗^, p(20–28 hr)^∗∗∗^, p(prepupal–20 hr)^∗∗∗^; whereas it is similar for less-bright regions: p(prepupal–28 hr)^∗^, p(20–28 hr)NS, p(prepupal-20 hr)NS. (L) *ActP-fz-EYFP/ptc-GAL4, UAS-shi^ts1^* in prepupal wings. Dotted lines are FRAP on tissue outside the *ptc-GAL4* domain in the same wings, for comparison. The unstable fraction in both bright and less-bright regions is significantly increased in mutant compared to wild-type (p^∗∗∗^). The half-life of recovery is not significantly different for bright regions (wild-type, 20.44 s; mutant, 25.18s; p = 0.39) or less-bright regions (wild-type, 22.85 s; mutant, 19.82 s; p = 0.69). See also Figure S5.

Staining for total Fmi in 5.5- and 20-hr wings also showed core protein colocalization in junctional puncta (Figures 3L, 3M, 4C, and 4D). However, at 20 hr APF, when cells are undergoing junctional remodeling, asymmetric localization is poor (Classen et al., 2005; Aigouy et al., 2010), and puncta are smaller than at 28 hr (Figure 3Q). Notably, the loss of cellular asymmetry seen in *dgo*, *pk*, or *dsh* mutant tissue also results in the core proteins forming progressively smaller puncta of slightly reduced intensity (Figures 3N–3R, 6F, and 6G; Figures S4B–S4M), correlating with the strength of polarity defects seen in these backgrounds (see Strutt and Strutt, 2007).

Overall, we find that in addition to an unstable population of Fmi that is subject to endocytosis and recycling, there is a population that is apparently refractory to turnover and localized to junctional puncta that are the major sites of asymmetry. Time-lapse imaging of Fz-EYFP demonstrates that such puncta persist over several hours (Figure 3S and Movie S1).

### FRAP Reveals Stable Fractions of Core Proteins in Puncta

To distinguish whether proteins within puncta are stably localized, or if puncta are persistent but the protein content changes, we used FRAP in pupal wings to measure turnover in real time. We employed two transgenes, one expressing *fz-EYFP* under the *Actin5C* promoter (Strutt, 2001), and the other expressing *fmi-EGFP* under the *armadillo* promoter. Both proteins localize asymmetrically (Figures 4A and 4B) and rescue the mutant phenotype (Strutt, 2001; data not shown). Fz-EYFP is expressed at levels comparable to endogenous Fz (Figure S5A), whereas Fmi-EGFP is expressed at low levels that do not significantly increase total Fmi levels (Figure S5B).

During FRAP, up to 80% of the fluorescence in selected regions was bleached, without apparent detrimental effect on the tissue (Figures S5C and S5D). To minimize acquisition bleaching, images were collected at 30-s intervals (Figure S5E), and a pixel size of 139 nm was used, at the expense of reduced resolution (Figure 4A). Regions of fixed size enriched for bright fluorescence within apicolateral junctions were manually selected and compared with equivalent-sized regions not containing bright fluorescence. The selected bright regions were highly enriched for puncta and contained on average twice as much Fz-EYFP or Fmi-EGFP fluorescence as the less-bright regions but generally also included a small proportion of less-bright nonpuncta material. Conversely, the less-bright regions may contain small puncta that are below the limit of resolution (Figure 4A).

Strikingly, FRAP analysis of both Fz-EYFP and Fmi-EGFP showed that the bright regions within the junctions have a large stable fraction (low recovery) indicating that overall Fmi/Fz turnover is low, whereas the less-bright regions have a significantly smaller stable fraction (more recovery, Figures 4E and 4G, and Table 1). The remaining stable fraction in less-bright regions is likely to be in part due to the presence of stable accumulations of polarity proteins that are not large enough to be seen as visible puncta. In support of this there is an even smaller stable fraction of Fz-EYFP in anterior-posterior membranes, where no puncta are seen (Figure 4F and Table 1; p < 0.001 compared to bright and less-bright regions).

We also investigated the effects of altering the timing and levels of Fz expression. If Fz-EYFP expression was initiated only in larval stages rather than expressed throughout development, although no reproducible change in Fz levels was seen (Figure S5A), the stable fraction seen in less-bright regions was slightly reduced (Figure 4H and Table 1). When overall Fz levels were reduced by removing endogenous *fz*, the stable fraction of the less-bright regions decreased further (Figure 4I and Table 1). However, neither manipulation altered the stable fraction seen in puncta-rich bright regions. From this we conclude that the large stable fraction of Fz-EYFP seen in puncta-rich regions is not sensitive to timing or level of expression, nor is it affected by the presence or absence of endogenous Fz, and so probably reflects the normal behavior of Fz. However, “surplus” Fz can form stable complexes and accumulate in the less-bright regions between puncta.

Because the appearance of puncta and the degree of core protein asymmetry change over developmental time, we examined whether this correlates with the distribution of the stable fraction of Fz-EYFP. FRAP analysis of cultured prepupal wings showed that the bright regions again have a large stable fraction, whereas the less-bright regions have a smaller stable fraction (Figures 4C and 4J, and Table 1). Interestingly, at 20 hr APF, when there is low asymmetry (Figure 4D), FRAP reveals that a sizeable stable fraction of Fz-EYFP is still present, but this is no longer concentrated in bright regions (Figure 4K and Table 1).

### The Unstable Fraction of Fz-EYFP Is Subject to Endocytic Trafficking and Lateral Diffusion

We next sought to determine if endocytosis and recycling affect the recovery of the unstable fraction of Fz-EYFP in FRAP experiments. We predicted that if unstable material was normally removed from junctions by endocytosis, then expressing dominant-negative dynamin at the restrictive temperature would lead to an accumulation of excess unstable material. Consequently, FRAP analysis would reveal a relatively larger unstable fraction, and this is what we observe in both bright and less-bright regions (Figure 4L).

However, blocking endocytosis did not cause any measurable change in the rate of recovery of fluorescence (Figure 4L), and this was also true if recycling was blocked (data not shown). A caveat to this result is that the half-life of recovery in both cases is similar to the sampling interval, precluding accurate measurement (see Experimental Procedures). Nevertheless, these observations suggest that endocytosis/recycling are not important for the rapid recovery of the unstable fraction and, instead, are consistent with lateral diffusion being the primary mechanism.

### Fmi Is Stabilized at Junctions by the Transmembrane Core Proteins

Our data so far have demonstrated that a stable population of Fmi and Fz localizes to junctions and is concentrated in puncta. Antibody internalization also reveals a labile population of Fmi in the apical membrane, apparently not associated with Fz or Stbm. We hypothesize that Fmi is normally trafficked to an apical/junctional plasma membrane compartment, where it undergoes rapid endocytosis, and Fz and Stbm serve to stabilize Fmi in the apicolateral junctional region. To test this we measured Fmi internalization in the absence of Stbm, Fz, or both: according to our model, this should cause a progressive increase in the rapidly endocytosed population of Fmi.

**Figure 5:**
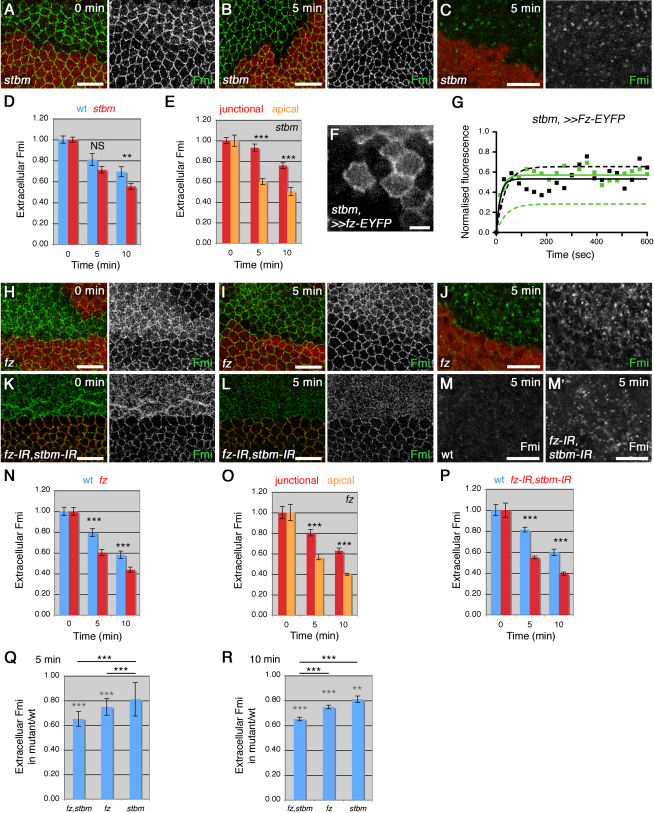
Fz and Stbm Stabilize Junctional Fmi Fmi antibody internalization (A–E and H–R) or Fz-EYFP FRAP (F and G) in mutant backgrounds. (A–C and H –M) Images of Fmi antibody internalization in *stbm^6^* clones (A–C), *fz^P21^* clones (H–J), and *ptc-GAL4, UAS-fz-IR,UAS-stbm-IR* (K–M), mutant tissue marked by loss of β-gal (red, A–C and H –J) or loss of Stbm (red, K and L). (A, H, and K) Extracellular Fmi (green) with 0-min chase. (B, I, and L) Extracellular Fmi (green) with 5-min chase. (C, J, and M) Total Fmi staining with 5-min chase, subapical sections. (M) and (M′) show Fmi staining in wild-type and *fz,stbm* mutant regions of the same wing. Steady-state levels of Fmi are increased in mutant tissue (1.8-, 2.2-, and 2.0-fold for *stbm*, *fz*, and the double mutant, respectively). Scale bars, 10 μm. (D, E, and N–R) Quantitation of extracellular staining. (D, N, and P) Total extracellular staining, asterisks indicate p values of mutant relative to wild-type at the same time point. Because absolute internalization varied significantly between experiments (Figure S1A), mutant and wild-type tissue within the same wings was compared. (E and O) Junctional or apical extracellular populations of Fmi. Asterisks indicate p values comparing junctional and apical Fmi. (Q nd R) Relative internalization of Fmi in mutant relative to wild-type tissue, at 5- and 10-min chase times. Gray asterisks indicate p values relative to wild-type at the same time point; black asterisks indicate p values comparing genotypes as indicated by the bar. Image (F) or FRAP analysis (G) on live wings of genotype *Ubx-FLP; ActP-FRT-polyA-FRT-fz-EYFP*, *stbm^6^/stbm^6^*. (F) Scale bar 5 μm. (G) FRAP on bright (green) or less-bright (black) regions; dotted lines indicate 28-hr wing data for *Ubx-FLP; ActP-FRT-polyA-FRT-fz-EYFP/+* for comparison (see Figure 4H). See also Figures S1–S3.

In *stbm* mutant tissue, extracellular Fmi is moderately well localized to junctions, but no clear puncta are present, and there is a slight increase in the apical population (Figure 5A; Strutt and Strutt, 2008). Overall, there is more rapid internalization of extracellular Fmi staining in *stbm* compared to wild-type tissue, and a corresponding increase in the number of intracellular Fmi structures (assumed to be endosomes, Figures 5A–5D). As in wild-type tissue, apical Fmi is internalized more rapidly than junctional Fmi (Figures 3A and 5E), indicating that a fraction of Fmi is still being stabilized in junctions. However, the rate of internalization of junctional Fmi in *stbm* is similar to that of nonpuncta Fmi in wild-type (∼80% in 10 min, Figures 3A and 5E). The absence of a stable protein population concentrated in junctional puncta in *stbm* tissue was confirmed by FRAP: although regions of varying Fz-EYFP brightness can be seen in junctions (Figure 5F), there is a similar stable fraction regardless of the brightness (Figure 5G and Table 1).

Loss of *fz* function causes a more severe loss of extracellular Fmi at junctions and a larger apical population: again, an increase in Fmi internalization was seen (Figures 5H–5J and 5N). Comparing the ratio of extracellular Fmi in mutant relative to wild-type tissue indicated that more Fmi was internalized in *fz* mutant tissue than in *stbm* mutant tissue during the first 5 min (Figure 5Q). Again, apical Fmi is internalized more rapidly than junctional Fmi (Figure 5O), supporting the idea that Fz and Stbm each play a role in stabilizing Fmi at junctions.

In tissue lacking both *fz* and *stbm*, where Fmi is almost entirely localized apically, overall Fmi internalization was very rapid (Figures 5K–5M and 5P–5R; Figures S2F–S2K; see also Figures S1D and S1F–S1H). Notably, the overall internalization is very similar to that of the apical Fmi population alone in wild-type, or in *fz* or *stbm* single mutants (∼60% in 5 min, Figures 3A, 5E, 5O, and 5P). Internalization of the apical Fmi occurred most quickly within the first 5 min, but subsequently, extracellular Fmi levels were reduced more slowly (Figure 5P), probably because Fmi is already being recycled back to the apical surface: in *fz,stbm* mutant tissue we see colocalization of internalized Fmi vesicles with Rab11, as well as with Rab5 and Rab7 (Figures S3J–S3L).

Although internalization is increased in these mutant backgrounds, overall steady-state levels of Fmi at the cell surface are also increased, suggesting that increased internalization is balanced by increased delivery of Fmi to the plasma membrane. Altering the overall levels of Fmi at junctions does not alter its rate of internalization (Figure S1L), suggesting that the shift from junctional to apical localization, rather than the change in levels, is responsible for increasing Fmi endocytosis.

### Cytoplasmic Core Proteins Are Required for Concentration of Fz/Fmi in Puncta

We have shown that Fz and Fmi stably localize to junctions, and that Stbm enhances this stability and is also required for concentration of a stable fraction in puncta. We next investigated if the cytoplasmic core proteins have any role in formation of a stable junctional fraction. Puncta are smaller, and core protein asymmetry is reduced in *dgo*, *pk*, and *dsh* mutant backgrounds (Figures 3N–3Q). This may indicate that the cytoplasmic proteins are required for formation of stable complexes, or alternatively, they may be required for the concentration of stable complexes into puncta.

**Figure 6:**
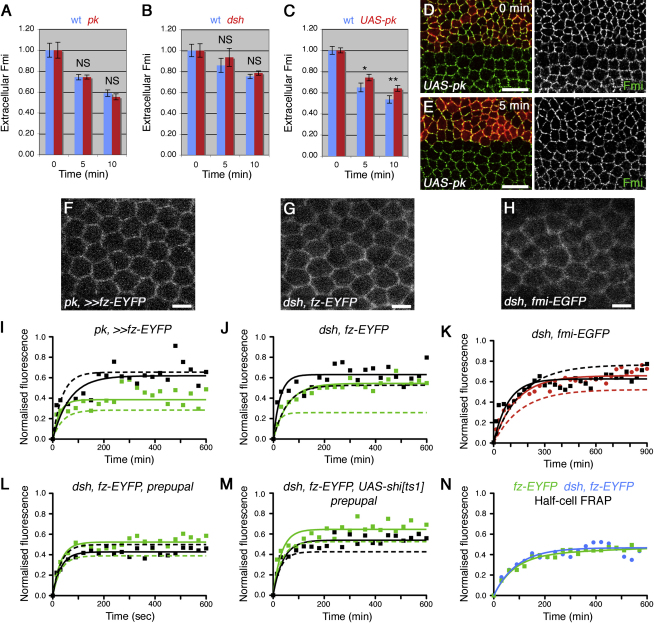
Cytoplasmic Core Proteins Concentrate Fz and Fmi into Puncta (A–C) Quantitation of Fmi antibody internalization in *pk^pk-sple13^* clones (A), *dsh^V26^* clones (B), and *ptc-GAL4, UAS-pk* (C). Asterisks indicate p values of mutant relative to wild-type at the same time point. (D and E) Fmi antibody internalization in *ptc-GAL4, UAS-pk*, mutant tissue marked by Pk (red). Extracellular Fmi staining (green) with 0-min (D) or 5-min (E) chase. Scale bars, 10 μm. (F–H) *Ubx-FLP; ActP-FRT-polyA-FRT-fz-EYFP, pk^pksple13^/pk^pksple13^* (F), *dsh^1^; ActP-fz-EYFP/+* (G), or *dsh^1^; ArmP-fmi-EGFP/+* (H) live 28-hr wings. Scale bars, 5 μm. (I–K) FRAP analysis on bright regions (green) or less-bright regions (black) of wings of genotype *Ubx-FLP; ActP-FRT-polyA-FRT-fz-EYFP, pk^pksple13^/pk^pk-sple13^* (I), *dsh^1^; ActP-fz-EYFP/+* (J), and *dsh^1^; ArmP-fmi-EGFP/+* (K). Dotted lines indicate 28-hr data for *Ubx-FLP; ActP-FRT-polyA-FRT-fz-EYFP* (I), *ActP-fz-EYFP/+* (J), and *ArmP-fmi-EGFP/+* (K) for comparison (see Figures 4H, 4E, and 4G). (I and J) p values comparing the stable fractions of bright regions for Fz-EYFP are: p(wt-*pk*)^∗∗∗^, p(wt-*dsh*)^∗∗∗^, p(*pk-dsh*)^∗∗∗^, and less-bright regions are not significantly different from wild-type wings. (K) The stable fraction of Fmi-EGFP in bright and less-bright regions is significantly different in wild-type compared to *dsh* mutant wings (p^∗∗∗^). (L and M) FRAP analysis on *dsh^1^; ActP-fz-EYFP/ptc-GAL4; UAS-shi^ts1^*/+ prepupal wings. (L) FRAP on tissue outside of the *ptc-GAL4* domain; dotted lines show FRAP on nonmutant *ActP-fz-EYFP* wings raised at the same conditions for comparison (see Figure 4L). The stable fraction in bright (green) and less-bright (black) regions is significantly different in wild-type compared to mutant tissue (p^∗∗∗^). Note that bright regions now have a smaller stable fraction than less-bright regions, indicating that the residual bright regions are not clusters of stable protein. (M) FRAP on tissue in the *ptc-GAL4* domain; dotted lines indicate data for *dsh^1^; ActP-fz-EYFP*/+ tissue in same wings for comparison (see L). The unstable fraction in bright and less-bright regions is significantly increased in *dsh, shi* compared to *dsh* (p^∗∗∗^). (N) Overall recovery of fluorescence after half cell bleaching, in *ActP-fz-EYFP/+* (green) or *dsh^1^; ActP-fz-EYFP/+* (blue) wings. The stable fraction for *ActP-fz-EYFP/+* (0.46) is not significantly different from the stable fraction for *dsh^1^; ActP-fz-EYFP/+* (0.47; p = 0.44).

We first tested whether cytoplasmic components affect rate of turnover of cell surface Fmi but found no alteration in internalization in *dsh* or *pk* mutants (Figures 6A and 6B). Notably, overexpression of any of the cytoplasmic components (Pk, Dsh, and Dgo) causes excess accumulation of the other core proteins in large junctional puncta (Feiguin et al., 2001; Tree et al., 2002; Bastock et al., 2003), and this causes a reduction in apical Fmi (Figure 6D) and a small (but significant) reduction in Fmi internalization (Figures 6C–6E). These results suggest that cytoplasmic proteins do not normally affect turnover of Fmi but when overexpressed can shift an unstable fraction into a stable fraction, concentrated in abnormally large puncta.

To test for a general role of Pk and Dsh in concentrating proteins in puncta, we carried out FRAP experiments. In *pk* mutants at 28 hr, there is a loss of polarity, and puncta are smaller than in wild-type (Figures 3O and 6F): here, the stable fraction of Fz-EYFP in the bright regions decreases compared to wild-type but is still larger than in the less-bright regions (Figure 6I and Table 1). *dsh* mutants have a stronger loss of polarity (see Strutt and Strutt, 2007), and the stable fraction in the bright regions decreases further for both Fz-EYFP and Fmi-EGFP at 28 hr (Figures 6G, 6H, 6J, and 6K, and Table 1), with an even more pronounced effect at 5.5 hr (Figure 6L).

Because in *pk* and *dsh* mutants the rate of Fmi internalization is not altered (Figures 6A and 6B), the decreased stable fraction seen in bright regions upon FRAP in these backgrounds (Figures 6I–6L) is unlikely to be due to a change in rate of endocytosis of either stable or unstable fractions of Fmi/Fz. Rather the stable fraction may simply be dispersed within junctions. In support of this, expression of dominant-negative dynamin in a *dsh* mutant caused an additive rather than a synergistic effect on the proportion of unstable Fz-EYFP: there was a slight increase in the unstable fraction in *shi,dsh* tissue, compared with *dsh* alone (Figures 6L and 6M), which was similar to the increase seen in *shi* alone (Figure 4L).

To test directly if the stable fraction was dispersed, we bleached the junctions throughout half a cell and measured the recovery of the entire region. Strikingly, the overall stable fraction of Fz-EYFP was similar in a wild-type and a *dsh* mutant background (Figure 6N). Thus, our data together suggest that the role of the cytoplasmic core proteins is not to generate a stable fraction of Fz/Fmi but to concentrate it within discrete membrane subdomains.

## Discussion

### Asymmetric Protein Complexes and the Establishment of Planar Polarity

Since the first report over a decade ago of the asymmetric subcellular localization of Fmi in the *Drosophila* pupal wing (Usui et al., 1999), the mechanisms underlying the distribution of the core polarity proteins have been extensively investigated (reviewed in Strutt and Strutt, 2009). A growing number of models have been presented to describe how the core proteins might achieve asymmetric localization (e.g., Amonlirdviman et al., 2005; Klein and Mlodzik, 2005; Le Garrec et al., 2006), with a common feature being the general assumption that the core proteins assemble together into a stable asymmetric intercellular complex. However, the existence of such a complex is largely inferred from the distributions of the proteins, and the actual roles of individual proteins in the formation, stabilization, and subcellular distribution of such complexes are poorly understood.

We show here that a fundamental organizing principle for core protein asymmetry is their distribution into discrete plasma membrane subdomains in the apicolateral junctions, which we refer to as “puncta.” Using the independent methodologies of antibody internalization and FRAP, we demonstrate that the populations of Fmi and Fz in puncta are highly persistent, supporting the view that the core polarity proteins do indeed form stable asymmetric complexes, and that these complexes are preferentially clustered together in puncta.

Our data allow us to make several inferences about the formation of such asymmetric complexes. We previously observed that in the absence of Stbm, an asymmetric Fz-Fmi:Fmi complex was preferentially formed between neighboring cells (Strutt and Strutt, 2008). Our results suggest that this Fz-Fmi:Fmi complex is the primary building block for the core protein complex. In the absence of Fmi, Fz does not localize to junctions (Strutt, 2001), and in the absence of Fz, Fmi is also poorly localized to junctions (Usui et al., 1999; Strutt and Strutt, 2008) and subject to endocytic turnover (this work). Importantly, loss of other core proteins (Stbm, Pk, Dsh) has less or no effect on Fmi endocytosis, and similarly does not eliminate the stable fraction of Fz, indicating that Fz and Fmi stably localize to junctions in the absence of these factors. Nevertheless, although Stbm does not preferentially form an asymmetric complex with Fmi in the absence of Fz (Strutt and Strutt, 2008), its ability to further stabilize Fmi at junctions in the presence of Fz indicates an important secondary role in formation of the asymmetric complex.

Although the cytoplasmic core proteins do not appear to play any role in the formation of stable complexes, they do promote the “clustering” of such complexes into puncta. This is consistent with previous data suggesting that the cytoplasmic factors are not required for intercellular communication but that they have an intracellular function in generating asymmetry (Strutt and Strutt, 2007; Chen et al., 2008). This absence of a requirement for the cytoplasmic factors in polarized intercellular communication, and the ability of Fz and Fmi to form asymmetric complexes in the absence of Stbm (Strutt and Strutt, 2008) both suggest that protein complexes are already asymmetric in the absence of clustering.

Several lines of data suggest that puncta are functionally important for generation of cellular asymmetry. First, they are the major sites of asymmetric localization of the core proteins. Second, their size, and the degree to which they contain a stable faction of Fz, varies over time and correlates with the degree of cellular asymmetry observed. Third, core polarity gene mutations that affect cellular asymmetry to different extents have a corresponding effect on the size of the stable fraction of Fz in puncta.

The mechanism by which asymmetric complexes are clustered into puncta is unknown. The simplest model is that cytoplasmic factors act as “glue” to hold complexes of the same orientation together and reduce their rates of lateral diffusion in the membrane. The alternative hypothesis that the cytoplasmic factors promote clustering by reducing rates of endocytic turnover is inconsistent with our observation that the overall stable fraction is not altered in the absence of cytoplasmic core protein function. The preference for clustering complexes of the same polarity may also be promoted by inhibitory interactions between proximal and distal complex components (Tree et al., 2002; Jenny et al., 2003, 2005; Das et al., 2004).

A key question is how such clustering might lead to the establishment of cellular asymmetry. One possibility is a process of self-organization involving local self-enhancement and longer-range inhibition (Turing, 1952; Gierer and Meinhardt, 1972). If planar polarity represents such a self-organizing process, clustering of asymmetric complexes into puncta is likely to provide local enhancement, whereas formation of intrinsically asymmetric complexes between cells may effectively provide longer-range subcellular inhibition that prevents all the clusters within a cell having the same orientation (Meinhardt, 2007). In support of such self-organization in the pupal wing, we note that induction of Fz, Fmi, or Stbm expression as late as 24 hr APF can lead to locally organized cellular polarity within a few hours (Strutt and Strutt, 2002, 2007) that is not oriented on the PD axis and as such is unlikely to be specified by long-range patterning cues.

**Figure 7:**
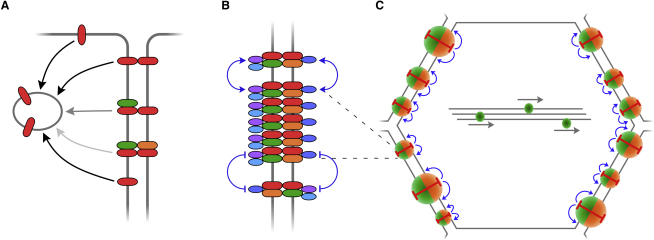
Model Showing Progressive Organization of Core Proteins (A) Fmi (red) localized apically or laterally is rapidly endocytosed (black arrows), unless it is in an asymmetric junctional complex with Fz (green) or Fz and Stbm (orange). (B) Cytoplasmic proteins Dsh and Dgo (purple and pale blue) or Pk (dark blue) are incorporated into asymmetric complexes, and promote local clustering of complexes of the same orientation by either attractive homophilic interactions or repulsive heterophilic interactions (blue lines and arrows). (C) Locally organized clusters of asymmetric complexes form distinct “puncta” in cell-cell junctions. Cellular asymmetry may be promoted by self-organization of asymmetric complexes within the cell. Individual puncta containing complexes of common polarity increase in size by recruiting further complexes of the same polarity and/or repelling complexes of opposite polarity, leading to local self-enhancement of asymmetric protein distribution (blue arrows). Because intercellular complexes are intrinsically asymmetric, local self-enhancement of Fz clustering in one cell leads to a corresponding enhancement of Stbm clustering in the neighboring cell. This coupled clustering acts as a form of long-range intercellular inhibition (red bars), ensuring that within each cell, domains containing high concentrations of both Fz and Stbm form. Global orientation of such clusters relative to the axes of the tissue is specified by long-range cues, such as distal transport of Fz-containing vesicles (center of cell; Shimada et al. [2006]; Harumoto et al. [2010]).

Overall, we propose a model in which molecular asymmetry is initially established by formation of Fz-Fmi:Fmi complexes that are intrinsically stable and in which Fmi endocytosis is attenuated (Figure 7). Entry of Stbm into the complex further promotes Fmi localization to junctions. The cytoplasmic components Dsh, Pk, and Dgo can then be recruited into the complex but do not increase its stability. Instead, they are required for clustering of asymmetric complexes of common polarity into junctional puncta, which are sites of local asymmetry. Through a self-organization process, which would normally be globally biased by an upstream patterning cue, locally organized puncta adopt an asymmetric distribution within the cell, linking the polarity of neighboring cells.

### Puncta and the Remodeling of Planar Polarity

Our time-lapse experiments indicate that individual puncta are stable for several hours (see also Aigouy et al., 2010). Nevertheless, in the *Drosophila* wing, morphogenetic changes such as wing eversion, hinge contraction, and junctional remodeling (Classen et al., 2005; Aigouy et al., 2010) necessitate some rearrangement of junctions, and this appears to be accompanied by reduced puncta size and loss of cellular asymmetry. Interestingly, although during junctional remodeling (at 20 hr APF), brighter regions are still visible in the junctions, our FRAP experiments reveal that these regions are no longer enriched for the stable fraction of Fz. This suggests that the membrane subdomains in which puncta form may be persistent, but the mechanisms that promote accumulation of asymmetric complexes in puncta are not active. This may allow the remodeling of planar polarity, following morphogenetic changes.

Transient asymmetric localization of polarity proteins is also seen in more dynamic systems, for example in vertebrate gastrulation (Ciruna et al., 2006; Yin et al., 2008), where their distribution is also highly punctate. It is possible that in cells that are undergoing movement and changing their contacts, local organization of polarity proteins into puncta allows more rapid reestablishment of polarized interactions between neighboring cells.

## Experimental Procedures

Additional information regarding fly stocks and antibodies is available in the Supplemental Experimental Procedures.

### Fly Genetics

Pupae were aged at 25°C for 5.5 hr for prepupal wings and 28 hr for pupal wings, unless otherwise indicated. Mitotic clones were induced using the FLP/FRT system (Xu and Rubin, 1993) and either *Ubx-FLP* (Emery et al., 2005) or *hs-FLP* (Golic and Lindquist, 1989). Overexpression used the GAL4/UAS system (Brand and Perrimon, 1993) with the *ptc-GAL4* driver. Larvae were aged at 19°C and shifted to 25°C at 0 hr APF for *UAS-Rab4^SN^* and *UAS-Rab11-IR*, or raised at 25°C and shifted to 29°C at 0 hr APF followed by aging for 4.25 hr for *UAS-pk* and *UAS-fz-IR*, *UAS-stbm-IR*. For overexpression or mitotic clones of *shi^ts1^*, larvae were aged at 19°C and prepupae shifted to 34°C 2 hr before dissection, or to 30°C for 30 min before imaging for FRAP. Expression of *Rab5^SN^* from the transgene *UHR-Rab5^SN^* was induced by crossing to *hs-FLP; ptc-GAL4* and subjecting prepupae to a 90-min heat shock at 38°C 2 hr before dissection.

### Immunostaining and Westerns

Prepupal wings were dissected at 5.5 hr APF, and pupal wings at 20 hr APF or 28 hr APF and imaged as previously described (Strutt, 2001). High-resolution images of fixed wings were taken on a Leica SP1 confocal microscope using a 100× NA1.4 oil apochromatic lens at 2× zoom, giving a pixel size of 45–50 nm.

For western blots, 28-hr pupal wings were dissected directly into sample buffer, and one pupal wing equivalent was loaded per lane.

### Antibody Internalization Experiments

5.5 hr APF prepupal wings were dissected in Schneider's Medium (SM; Invitrogen) containing 10% fetal bovine serum (FBS; Sigma) and transferred to a microtiter plate on ice. Medium was replaced with Fmi antibody diluted in SM/FBS, and wings were incubated at 4°C for 30 min. Wings were washed briefly in SM/FBS and chased in 1 ml SM/FBS at RT for various times. Endocytosis was stopped by pipetting wings into SM/FBS at 4°C for 5 min, and wings were then fixed in 4% paraformaldehyde/PBS for 15 min. For detection of extracellular Fmi, tissue was incubated in secondary antibody in the absence of detergent, and postfixed before adding other antibodies with detergent. For total Fmi staining, secondary antibody was added in the presence of 0.1% Triton X-100. Wings were mounted in Mowiol containing 2.5% DABCO. For control experiments with Dextran, tissue was incubated with Fmi antibody for 30 min at 4°C, chased for 5, 30, or 60 min at RT, and Dextran-Texas Red (MW 10,000, lysine fixable; Molecular Probes) was added for 15 min before fixation.

For quantitation of extracellular staining, at least ten wings were imaged from at least two experiments, using a Z-spacing of 150 nm and constant confocal settings. The average fluorescence intensity of wild-type or mutant regions was determined using ImageJ, and averaged for the three most strongly staining slices (corresponding to the adherens junctions). Laser-off background was subtracted, and the readings were normalized to 1.0 at t0. For comparison between genotypes, Fmi internalization was expressed as a ratio of extracellular Fmi in mutant/wild-type. Error bars represent standard error of the mean, and statistical significance was determined using unpaired Student's t tests (p^∗^ ≤ 0.05; p^∗∗^ ≤ 0.01; p^∗∗∗^ ≤ 0.001; NS, not significant p > 0.05).

Incubation at 4°C effectively blocked endocytosis because no intracellular Fmi vesicles were seen at the end of the antibody incubation nor was internalization of Dextran seen. Addition of FBS to the medium did not affect Fmi endocytosis (Figure S1K).

### Live Imaging and FRAP Analysis in Pupal Wings

APF pupae were aged at 25°C and dissected half an hour before imaging, adapting the method of Classen et al. (2008). A small piece of cuticle was removed from above the developing wing, and the pupa was inverted and mounted in a drop of Halocarbon 700 oil in a glass-bottomed dish (Iwaki). Pupae were retained after imaging, and >95% eclose, suggesting minimal tissue damage. Prepupal wings were dissected in SM/FBS (Classen et al., 2008) and placed in a glass-bottomed dish with ∼20 μl of SM/FCS surrounded by a gasket of parafilm. A round coverslip was then placed on top, and a ring of wet paper tissue was added to prevent evaporation. Alternatively, wings were placed in ∼5 μl SM/FCS containing 1% methylcellulose on a slide surrounded by Sellotape Diamond tape. A round coverslip was then placed on top and nail varnish applied to prevent evaporation—in this case, samples were imaged no more than 1 hr after dissection. FRAP analysis of trafficking mutants was carried out in prepupal wings because pupal wing tissue was unhealthy under the same conditions. Wild-type and mutant tissue from the same wings was analyzed.

Samples were imaged on an inverted Zeiss LSM 510 confocal microscope, with a Zeiss 63× NA 1.4 oil apochromatic objective lens at 2× zoom with the pinhole open to maximize light detected. The 488 nm Argon laser was used at an output of 20%, and a 505–550 nm band-pass filter was used for detection. Single images with no averaging were taken to reduce acquisition bleaching.

For FRAP, plasma membrane regions containing concentrated Fz-EYFP (bright regions) or diffuse Fz-EYFP (less-bright regions) were selected and bleached, using the 488 nm Argon laser at 100% and passing 20 times over a region of interest (ROI) of 2 μm^2^—this is smaller than half of the distal membrane in a *Drosophila* pupal wing cell and about twice as large as a typical punctum at 28 hr APF. The size of the ROI could not be reduced because tissue movement makes the ROI hard to track over time. No laser bleaching occurs outside the ROI, indicating that loss of fluorescence outside the ROI (but within the same cell) and rapid recovery inside the ROI are likely to be due to lateral protein diffusion (Figures S5F and S5G).

Three prebleach images were captured, as well as an immediate post-bleach image, and then an image was taken every 30 s for up to 40 min. Faster image acquisition was attempted, but this led to increased acquisition bleaching (see Figure S5E).

For data analysis, Volocity (v.4.4 Improvision) was used to manually reselect and quantify the fluorescence of at least ten of the 2 μm^2^ bleached regions at each time point, and laser-off background was subtracted. To measure acquisition bleaching, readings were collected from each of four bright and less-bright nonbleached control regions, 2 μm^2^ in size, at least two cells away. Data were then corrected for acquisition bleaching and normalized against an average of the prebleached values. Data were plotted on an XY graph in Prism (v.5 GraphPad), and a one-phase exponential association curve was fitted. An extra sum-of-squares F test was performed to compare curve plateaux (Y^max^). Note that in most cases, the half-life of recovery was less than the acquisition interval (30 s), and thus, the rate of recovery could not be accurately determined.

### Imaging and Analysis of Puncta

For analyzing puncta size and intensity, samples were fixed, antibody stained, and imaged at maximum resolution (pixel size 47 nm), using identical settings. The *intermode* algorithm in ImageJ was used to select a suitable threshold value, which was then applied uniformly to all images being compared. The *particle analysis* tool was then used to define puncta with diameter larger than 240 nm, and to calculate the mean intensity and size of puncta. These conditions correctly identified all puncta that could be seen by eye but did not highlight endosomes, which were below the size cutoff. For antibody internalization a set threshold and particle size was selected for the 0-min images, and applied across images for later time points. Mean intensity, size, and number of puncta were determined. Nonpuncta junctional regions and apical regions were selected manually, and the mean intensity was measured.

For determining asymmetry in *fz-EYFP* and *stbm-EYFP* mosaics, puncta and nonpuncta regions on proximal and distal membranes were selected manually using Fmi staining. The mean intensity of GFP staining in regions of a fixed size was then determined.

## Figures and Tables

**Table 1:** Plateau Values for FRAP Experiments

Genotype	Y^max^				p Value
	Bright	95% Confidence Interval	Less Bright	95% Confidence Interval	Bright versus Less Bright
*ActP-fz-EYFP* 28 hr APF	0.26	0.25–0.27	0.53	0.50–0.56	≤0.001
*ActP-fz-EYFP* 28 hr APF (lateral)	–	–	0.72	0.68–0.75	–
*Ubx-FLP; ActP-FRT-polyA-FRT-fz-EYFP/+* 28 hr APF	0.28	0.26–0.31	0.64	0.59–0.69	≤0.001
*Ubx-FLP; ActP-FRT-polyA-FRT-fz-EYFP/+; fz^P21^* 28 hr APF	0.25	0.21–0.28	0.81	0.71–0.92	≤0.001
*ActP-fz-EYFP* prepupal 25°C^a^	0.34	0.32–0.37	0.66	0.60–0.72	≤0.001
*ActP-fz-EYFP* 20 hr APF	0.65	0.61–0.68	0.55	0.51–0.59	≤0.001
*ActP-fz-EYFP* prepupal 18°C/30°C^a^	0.39	0.38–0.40	0.50	0.48–0.53	≤0.001
*ActP-fz-EYFP/ptc-GAL4, UAS-shi^ts1^* prepupal 18°C/30°C	0.56	0.54–0.58	0.65	0.62–0.67	≤0.001
*Ubx-FLP; ActP-FRT-polyA-FRT-fz-EYFP*, *stbm^6^/stbm^6^* 28 hr APF	0.57	0.54–0.59	0.54	0.49–0.59	NS
*Ubx-FLP; ActP-FRT-polyA-FRT-fz-EYFP, pk^pksple13^/pk^pk-sple13^* 28 hr APF	0.39	0.35–0.40	0.62	0.57–0.68	≤0.001
*dsh^1^; ActP-fz-EYFP/+* 28 hr APF	0.53	0.51–0.56	0.64	0.59–0.70	≤0.01
*dsh^1^; ActP-fz-EYFP* prepupal 18°C/30°C	0.53	0.51–0.54	0.43	0.40–0.45	≤0.001
*dsh^1^; ActP-fz-EYFP/ptc-GAL4, UAS-shi^ts1^* prepupal 18°C/30°C	0.65	0.63–0.67	0.54	0.51–0.57	≤0.001
*ArmP-Fmi-EGFP* 28 hr APF	0.52	0.49–0.55	0.75	0.71–0.80	≤0.001
*dsh^1^; ArmP-Fmi-EGFP* 28 hr APF	0.66	0.63–0.69	0.63	0.60–0.66	NS

NS, not significant.

a Animals raised at 25°C have different stable fractions in puncta and nonpuncta from those raised at 18°C and shifted to 30°C before dissection.

## Supplemental Information

Document S1. Five Figures and Supplemental Experimental Procedures

Movie S1. Time-Lapse Imaging of Puncta in Pupal Wings Time-lapse movie of live pupal wing expressing ActP-fz-EYFP at 27-29 hr APF. Images were taken at 5 min intervals for 2 hr. Circles mark individual puncta followed over time; puncta persist in junctions for the duration of the movie. See Figure S3 for stills from this movie.
