# Supplementary material for: Dynamics of Core Planar Polarity Protein Turnover and Stable Assembly into Discrete Membrane Subdomains
Source: Dev Cell. 2011 Apr 19;20(4):511–25. doi: 10.1016/j.devcel.2011.03.018 (PMC3094756; doi:10.1016/j.devcel.2011.03.018)
Supplement: Document S1. Five Figures and Supplemental Experimental Procedures [file mmc1.pdf]

Developmental Cell, Volume *20*

Supplemental Information

Dynamics of Core Planar Polarity Protein Turnover and Stable Assembly into Discrete Membrane Subdomains

Helen Strutt, Samantha J. Warrington, and David Strutt

Inventory of supplementary material:

Figure S1 related to Figures 1 and 5

Figure S2 related to Figures 1 and 5

Figure S3 related to Figures 2 and 5

Figure S4 related to Figure 3

Figure S5 related to Figure 4

Supplemental movie 1 related to Figure 3

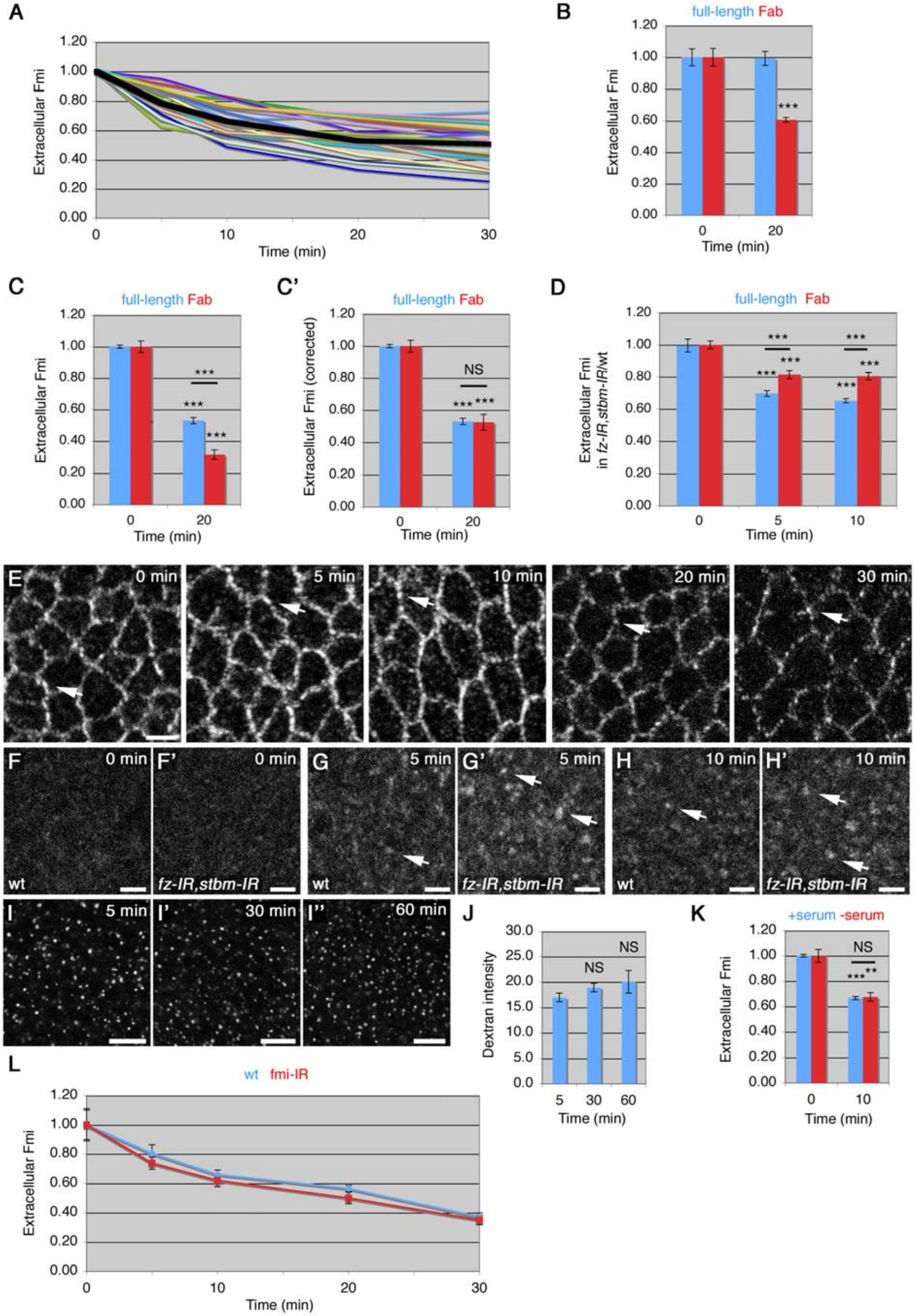

Supplemental Figure 1, related to Figures 1 and 5. Antibody internalisation controls.

(A) Quantitation of Fmi antibody internalisation with chase times up to 30 min in wildtype wings, in 19 independent experiments (coloured lines), and the mean internalisation (thick black line, as in Fig.1G).

(B) Quantitation of extracellular Fmi staining, after fixing wildtype prepupal wings for 5 min, incubating with full-length Fmi antibody or Fmi Fab antibody fragments for 30 min at 4°C, and chasing for 0 or 20 min. Stars indicate P values relative to 0 min. No full-length antibody falls off (blue), but 40% of Fab fragments have dissociated (red). A similar level of dissociation is seen in *fz,stbm* mutant tissue (not shown).

(C,C') Fmi antibody internalisation experiment in wildtype wings with a 0 min or 20 min chase, using full-length Fmi antibody (blue) or Fmi Fab antibody fragments (red), showing quantitation of extracellular Fmi staining. Stars indicate P values relative to 0 min, or as indicated by the bar below. (C) Raw data, indicating a greater reduction in Fmi staining using Fab fragments. (C') Data corrected for the antibody dissociation quantified in B. Internalisation of full-length Fmi antibody and Fmi Fab antibody fragments is similar.

(D) Quantitation of Fmi antibody internalisation experiment in *ptc-GAL4, UAS-fz-IR,UAS-stbm-IR* wings using full-length (blue) or Fmi Fab antibody fragments (red), at 0, 5 and 10 min chase times. Data show relative levels of extracellular Fmi in mutant compared to wildtype tissue of the same wings. Stars indicate P values relative to wildtype at the same timepoint, or as indicated by bars. Fmi is internalised faster in *fz,stbm* mutant tissue using both antibodies; however the effect is significantly stronger using full-length Fmi antibody. Note that in this experiment, as mutant and wildtype values are ratioed, this should provide an internal correction for fall-off of the Fab antibody fragment. However, the apparently slower rate of internalisation seen with the Fab fragments may be an artefact caused by mutant tissue having a greater fraction of internalised Fab fragment than wildtype tissue, and this internalised fraction being protected from dissociating from the tissue when in endosomes and then being recycled to the plasma membrane.

(E) Fmi antibody internalisation experiment in wildtype wings using Fmi Fab antibody fragments, stained for extracellular Fmi at various chase times. Although confocal settings are constant, confocal detector background is more prominent at later timepoints, as Fab antibody fragments fall off and the remaining population becomes increasingly difficult to detect. Nevertheless, a punctate junctional Fmi population (arrows) remains at junctions at 30 min, demonstrating that antibody cross-linking is not the cause of the failure of punctate Fmi to internalise. Scale bar 2.5 μm.

(F-H) Fmi antibody internalisation experiment in *ptc-GAL4, UAS-fz-IR,UAS-stbm-IR* wings using Fmi Fab antibody fragments, panels show wildtype and mutant tissue from the same wings for comparison. Panels show subapical sections of wings stained with total secondary antibody at 0 min (F), 5 min (G) and 10 min (H) chase times, using the same confocal settings. Puncta are difficult to visualise as Fmi Fab fragment staining is poor and antibody dissociates over time, however puncta can be seen in both wildtype and mutant tissue (arrows), and are more frequent in the latter. Scale bars 2.5 μm.

(I) Subapical sections of Dextran staining in wildtype wings, incubated with Fmi antibody (not shown) for 30 min, washed and chased for 5 min (I), 30 min (I') or 60 min (I'') at RT, before the addition of Dextran-TexasRed for a further 15 min at RT. Dextran uptake is similar at all three timepoints, suggesting that endocytosis does not slow down over time. Scale bars 10 μm.

(J) Quantification of Dextran internalisation in the experiment in panel I, scale in arbitrary units, with confocal detector background subtracted.

(K) Antibody internalisation experiment in wildtype wings using Fmi antibody, incubations in Schneider's Medium +/- foetal bovine serum. Stars indicate P values relative to 0 min. Addition of serum to the medium does not stimulate Fmi internalisation.

(L) Antibody internalisation in wings expressing *ptc-GAL4, fmi-IR*. Extracellular Fmi levels are reduced to 30 % of wild type levels, but asymmetric localisation and trichome polarity is normal (not shown). There is no significant difference in internalisation compared to wildtype tissue in the same wings.

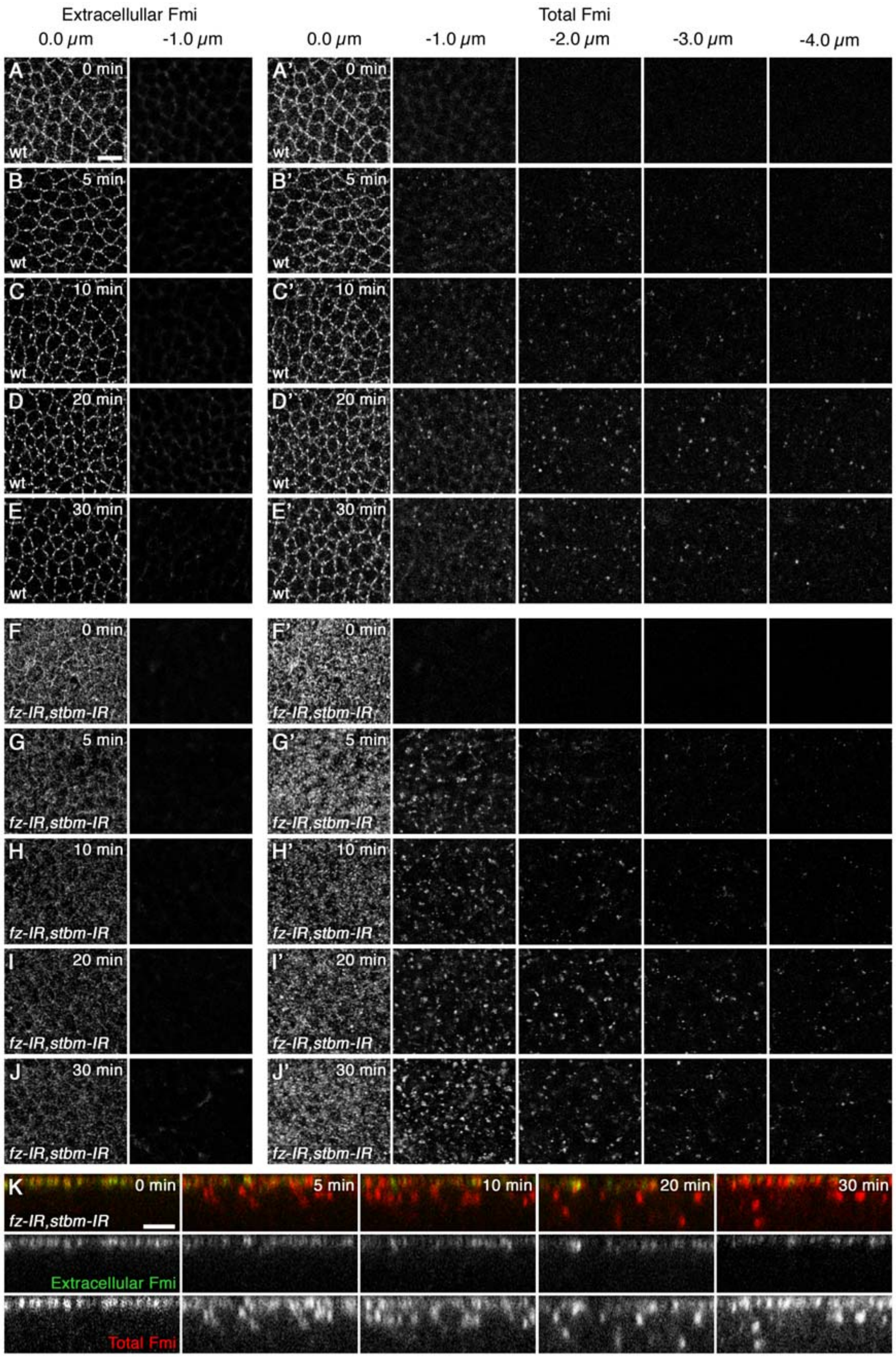

Supplemental Figure 2, related to Figures 1 and 5. Antibody internalisation in wildtype and *ptc-GAL4, UAS-fz-IR, UAS-stbm-IR* wings

Fmi antibody internalisation experiment in wild type (A-E) and *ptc-GAL4, UAS-fz-IR,UAS-stbm-IR* (F-K) wings. (A-J) Extracellular (left) and total (right) secondary antibody staining, apical to basal XY sections at the depths indicated, at the indicated chase times. Scale bars 5 μm. (K) XZ sections with extracellular (green) and total (red) secondary antibody staining. Scale bars 2.5 μm.

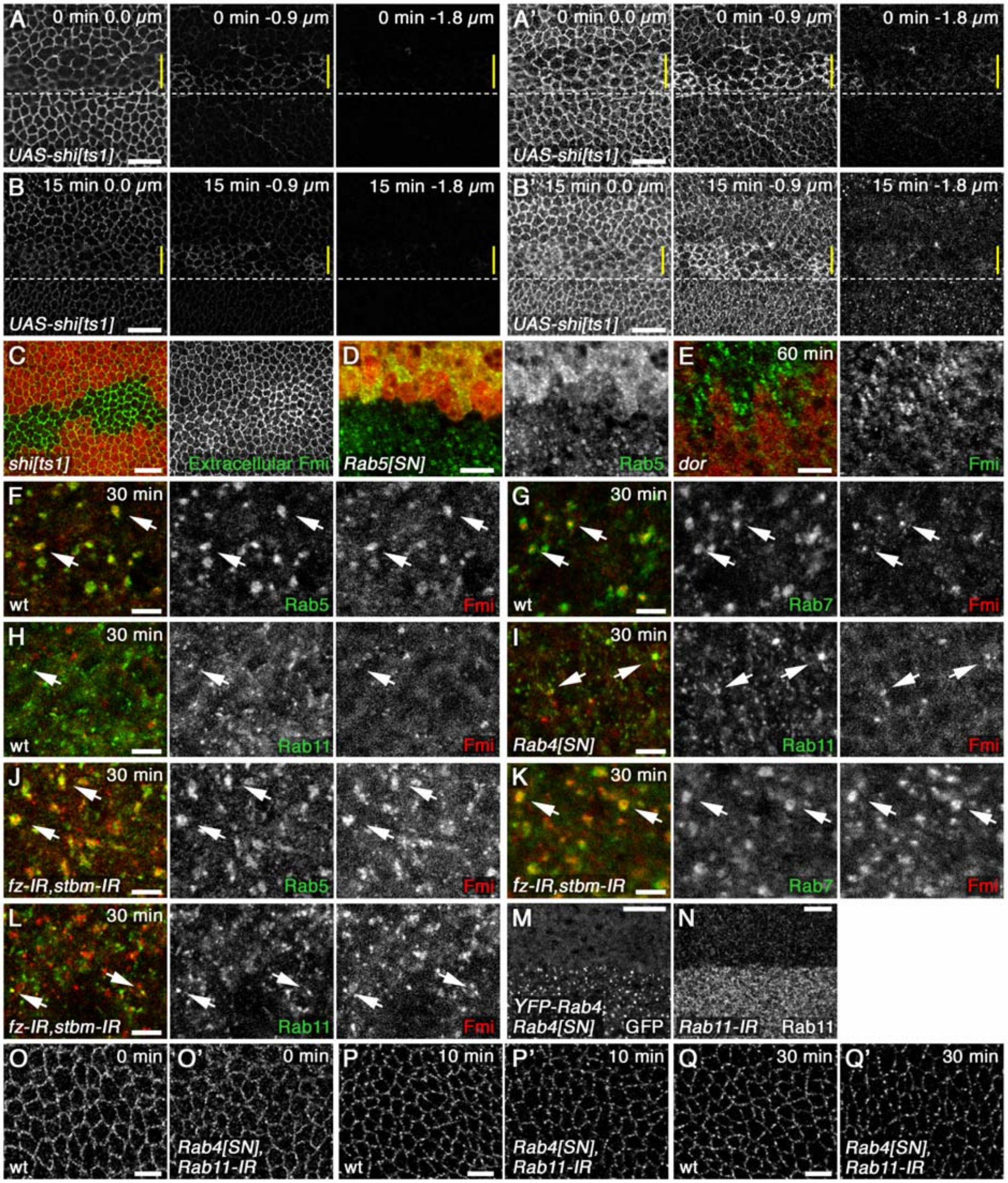

Supplemental Figure 3, related to Figures 2 and 5. Characterisation of Fmi trafficking pathways.

(A,B) Apical to basal XY sections at the depths indicated of extracellular (A,B) and total (A',B') Fmi staining in *ptc-GAL4, UAS-shi[ts1]* wings raised at 34°C for 2 hr before dissection, with a 0 min (A) or 15 min (B) chase at 34˚C, *ptc-GAL4* domain above the dotted line. In the most highly expressing region (yellow bar) cells are disrupted and junctional staining is seen in basal sections. Scale bars 10 μm.

(C) Extracellular Fmi staining (green) in live prepupal wing containing *shi*^{ts1} clones, marked by loss of β-gal (red), pupae shifted to 34°C for 2 hr prior to dissection. A 1.4-fold increase in extracellular Fmi is seen at junctions, compared to wildtype tissue. Scale bar 10 µm.

(D) Prepupal wings expressing *ptc-GAL4, UHR-Rab5*^{SN}, heatshocked 2 hr before dissection to excise the >DsRed> cassette and allow expression of *Rab5*^{SN}. The *ptc-GAL4* domain is marked by residual DsRed expression, here the Rab5 antibody recognises the dispersed dominant negative Rab5 protein (green), although some Rab5 positive puncta can still be seen. Expression of Rab5^{SN} for longer than 2 hr causes a failure in prepupal wing eversion (unpublished data). Scale bar 5 µm.

(E) Fmi antibody internalisation experiment, in prepupal wing containing *dor*^{8} clones, marked by loss of β-gal (red). Subapical sections with total Fmi staining (green) show accumulation of intracellular Fmi in vesicles after a 60 min chase. Scale bar 5 µm.

(F-L) Fmi antibody internalisation experiments, single subapical sections showing total Fmi staining after a chase time of 30 min. (F-H) wildtype, (I) *ptc-GAL4, UAS-Rab4*^{SN}, (J-L) *ptc-GAL4, UAS-fz-IR,UAS-stbm-IR wings*. Green is Rab5 (F,J), Rab7 (G,K), Rab11 (H,I,L), red is Fmi in all panels. Arrows indicate colocalising puncta. Scale bars 2.5 µm.

(M,N) Prepupal wings expressing *tub-YFP-Rab4; ptc-GAL4, UAS-Rab4*^{SN} (M) or *ptc-GAL4, UAS-Rab11-IR* (N). GFP staining (M) and Rab11 staining (N) are disrupted in the *ptc-GAL4* domain (top of panel), showing that *Rab4*^{SN} and *Rab11-IR* are effective. Note that expression of YFP-Rab4 in prepupal wings results in the appearance of YFP-Rab4 throughout the endocytic membranes (unpublished observations), so it cannot be used as a marker for the recycling compartment. Scale bars 10 µm.

(O-Q) Fmi antibody internalisation experiment in prepupal wings expressing *ptc-GAL4, UAS-Rab4*^{SN}*, UAS-Rab11-IR*, chase times of 0 min (O), 10 min (P) and 30 min (Q). Extracellular Fmi staining in wildtype and mutant regions of the same wing. Note increased removal of Fmi from the cell surface when recycling is blocked. Scale bars 5 µm.

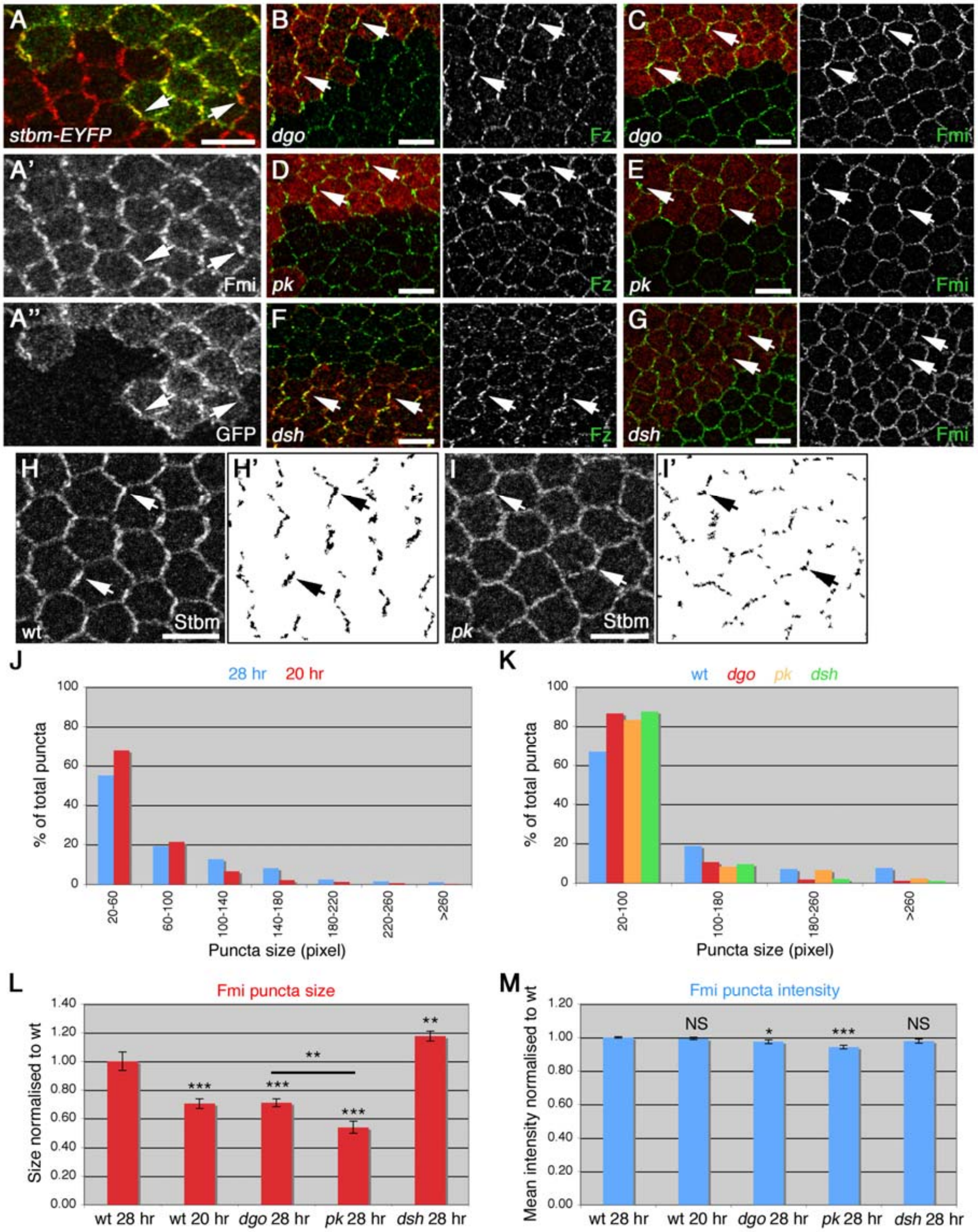

Supplemental Figure 4, related to Figure 3. Loss of large puncta in core polarity gene mutants

(A) Identification of the positions of puncta in *stbm-EYFP* mosaics (A'', green in A), using Fmi co-staining (A', red in A). Arrows mark two prominent Fmi puncta, one shows Stbm staining in puncta at the proximal cell edge, and the other is at the distal cell edge where Stbm staining is not concentrated in puncta.

(B-G) 28 hr pupal wings containing clones of *dgo*^{380} (B,C), *pk*$^{pk\text{-}sple13}$ (D,E) or *dsh*^{V26} (F,G), marked by loss of β-gal (red). Staining is Fz (green, B,D,F) or Fmi (green, C,E,G). Arrows point to puncta in wildtype tissue. Puncta are less prominent in mutant tissue.

(H,I) 28 hr pupal wing tissue showing Stbm staining in wildtype (H) or *pk*$^{pk\text{-}sple13}$ (I) tissue from the same wing. (H',I') The outcome of threshholding and particle analysis to identify puncta, using a constant threshhold in the wildtype and mutant areas. Arrows show prominent puncta, note puncta are overall smaller in the mutant (I).

Scale bars 5 μm.

(J,K) Distribution of puncta size in 28 hr and 20 hr pupal wings (J), or in wildtype and *dgo*^{380}, *pk*$^{pk\text{-}sple13}$ and *dsh*^{V26} mutant tissue (K), stained for Stbm. Puncta size is in pixels (1 pixel = 47 x 47 nm), and 20 pixels was the lower cut-off for puncta, corresponding to puncta with an average diameter of 240 nm. In (K) size categories are larger, to reduce noise due to the smaller sample size. More smaller puncta are seen at 20 hr and in the mutant conditions than in wildtype at 28 hr, at the expense of larger puncta. Distributions are significantly different using a chi-squared test for wildtype at 28 hr vs 20 hr (P***) and for wildtype vs *dgo* (P***), *pk* (P**) and *dsh* (P***) at 28 hr.

(L,M) Quantitation of puncta size (L) or mean fluorescence intensity (M) in 28 hr and 20 hr wildtype pupal wings, and in *dgo*^{380}, *pk*$^{pk\text{-}sple13}$ and *dsh*^{V26} mutant clones stained for Fmi. Stars are P values compared to 28 hr wildtype wings, or as indicated by bars. Note that, unlike the other core proteins, Fmi levels are slightly increased in *dsh* mutant tissue (Shimada et al., 2001; Strutt and Strutt, 2008), and thus the particle analysis failed to see a reduction in Fmi puncta size in these clones.

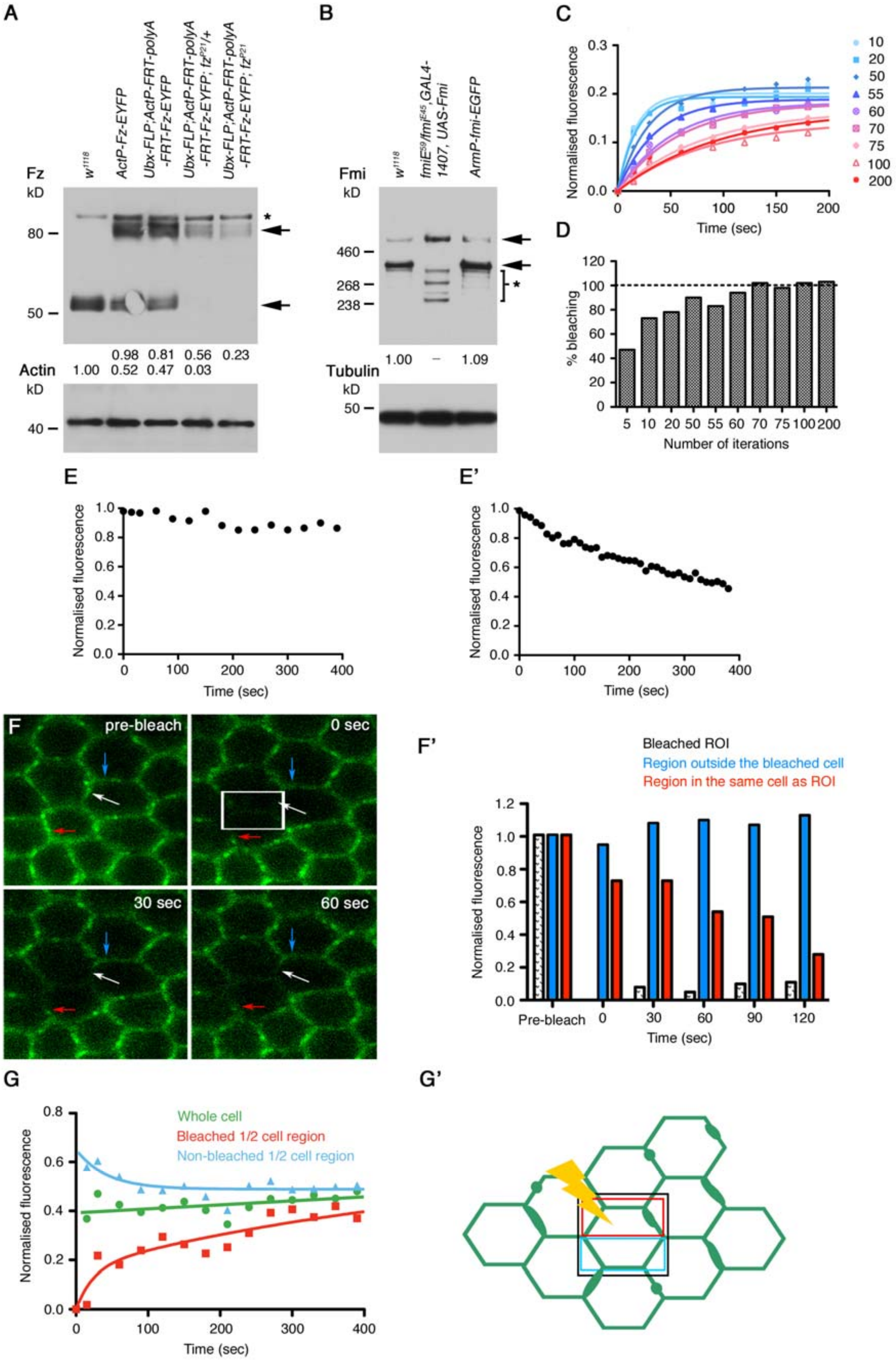

Supplemental Figure 5, related to Figure 4. Controls for FRAP experiments

(A) Western blot of 28 hr pupal wing lysates, probed with antibodies against Fz (top) followed by antibodies against Actin (bottom). The lower arrow indicates endogenous Fz, and the upper arrow indicates Fz-EYFP. The star indicates a non-specific band. Below is the quantitation of Fz-EYFP (top row) and Fz (bottom row) levels from three blots, after normalisation to the Actin loading control. The total amount of Fz plus Fz-EYFP is not significantly different (P=0.28) when *fz-EYFP* is expressed only in larval stages (genotypes containing *Ubx-FLP*) from when it is expressed throughout development (compare lanes 2 and 3). Note that Fz-EYFP levels are progressively and reproducibly reduced when *fz-EYFP* is expressed in the absence of one (lane 4) or both (lane 5) copies of endogenous *fz*. Additionally, when one copy of endogenous *fz* is removed, little Fz is detected (lane 4, some Fz is seen at longer exposures). Hence, increased levels of endogenous Fz result in higher levels of Fz-EYFP, suggesting that the proteins mutually stabilise each other.

(B) Western blot of 28 hr pupal wing lysates, probed with antibodies against Fmi (top) and a-tubulin (bottom). Note full-length and cleaved forms of Fmi (upper and lower arrows). The cleaved form of Fmi and Fmi-EGFP is the same size, as the C-terminal GFP tag is cleaved off. The mutant lane is the truncation allele fmi^{E59} over the missense allele fmi^{E45}, rescued through embryogenesis using *UAS-fmi* under control of *GAL4-1407* (see Usui et al., 1999). fmi^{E45} produces full-length protein, but it is not correctly processed, and a ladder of smaller bands is seen (star). Quantitation of the cleaved form of Fmi is shown.

(C) Graph of recovery of Fz-EYFP fluorescence after different amounts of bleaching. Bleaching was varied by altering the number of laser passes (iterations) over the bleached region, with laser power remaining constant (this method also varies the length of bleaching time, 1 pass = 1.2 sec). A similar percentage recovery is seen with up to 50 iterations, but recovery is reduced with more iterations, suggesting that above 50 laser passes the laser-exposure may induce cross-linking of proteins and thus inhibit fluorescence recovery.

(D) Graph showing the percentage of bleaching relative to the number of bleach iterations. A bleaching amount of between 50 and 80% is considered optimal to prevent artefacts occurring in the resulting data, as this should give a good signal-to-noise ratio without damage to the tissue.

(E) Graphs showing acquisition bleaching at 30 sec (E) and 10 sec (E') sampling intervals. Note that at a 30 sec acquisition interval, fluorescence does not fall below 80% of starting fluorescence during the course of the experiment (E), whilst using a 10 sec acquisition interval causes high acquisition bleaching (E').

(F,G) Experiments showing that lateral redistibution of fluorescence within junctional regions of the same cell, but not neighbouring cells, can account for early recovery in bleached regions.

Images (F) and quantitation (F'). (F) The area inside the white boxed area (ROI) was bleached. The white arrow indicates a region of membrane inside the bleached ROI, the blue arrow shows an unbleached region in a neighbouring cell membrane that shows no loss of fluorescence, and the red arrow indicates a region showing reduction in fluorescence outside the bleached ROI, but in a cell overlapping the ROI. (F') Fluorescence of the three regions indicated by the white, red and

blue arrows, measured over time in the same way as the other FRAP experiments. The fluorescence at the prebleach timepoint is normalised to 1 and the immediate postbleach timepoint (t0) is normalised to zero for the bleached region.

Quantitation (G) and diagramatic representation (G') of a half-cell bleaching experiment. Half a cell was bleached and three regions were measured for fluorescent intensity: the entire bleached half (red), the entire unbleached half (blue) and the whole cell (green). Recovery in the first minute is accompanied by loss of fluorescence from the unbleached half of the cell, whereas the whole cell measurement does not change significantly. Recovery approaches 40% consistent with half of the cell having been bleached.

Movie S1, related to Figure 3. Time lapse imaging of puncta in pupal wings

Time lapse movie of live pupal wing expressing *ActP-fz-EYFP* at 27-29 hr APF. Images were taken at 5 min intervals for 2 hr. Circles mark individual puncta followed over time; puncta persist in junctions for the duration of the movie. See Fig.3S for stills from this movie.

Supplemental Experimental Procedures

Fly stocks

Mutant alleles are described in FlyBase. Transgenes were *ActP-FRT-polyA-FRT-fz-EYFP* (Strutt, 2001) and the derivative *ActP-fz-EYFP*, *ActP-FRT-polyA-FRT-stbm-EYFP* (Strutt et al., 2002), *UAS-fz-IR* and *UAS-stbm-IR* (dsRNA lines in vector pWIZ, Bastock and Strutt, 2007), *UAS-pk* (Tree et al., 2002), *UAS-shi*^{ts1} (Kitamoto, 2001), *tub-YFP-Rab4* and *UHR-Rab5*^{SN} (Marois et al., 2006). *UAS-Rab4*^{SN} and *UAS-Rab11-IR* are derivatives of *UHR-Rab4*^{SN} and *FRIPE-Rab11* (Marois et al., 2006), in which the *FRT-DsRed-FRT* cassette was permanently excised. *ArmP-Fmi-EGFP* expresses GFP-tagged Fmi under control of the *armadillo* promoter, and *UAS-fmi-IR* contains a hairpin of bp 840-1763 (GenBank AB028498) in the first exon of the *fmi* coding sequence, in the pWIZ vector (Lee and Carthew, 2003).

Antibodies

Primary antibodies for immunostaining were mouse monoclonal anti-Fmi 74 (DSHB, Usui et al., 1999), rabbit anti-Fz (Bastock and Strutt, 2007), rat anti-Stbm (Strutt and Strutt, 2008), rat anti-Dsh (Strutt et al., 2006), rat anti-E-cadherin (DSHB, Oda et al., 1994), mouse monoclonal anti-Armadillo (DSHB, Peifer et al., 1994), rabbit anti-Rab5 (Abcam), rabbit anti-Rab7 (Tanaka and Nakamura, 2008), rabbit anti-Rab11 (Tanaka and Nakamura, 2008), mouse monoclonal anti-βgal (Promega), rabbit anti-βgal (Cappel) and rabbit anti-GFP (Abcam).

For live staining in antibody internalisation experiments, mouse monoclonal anti-Fmi 71 (Usui et al., 1999) was used. Fab fragments were generated by incubating 1 mg of purified IgGs with 10 μg papain in the presence of 100 mM sodium acetate pH5.2, 50 mM L-cysteine and 1 mM EDTA, for 8 hr at 37°C. Digestion was stopped by the addition of iodoacetate to 75 mM, and the buffer exchanged with PBS using a Vivaspin centrifugal concentrator (Vivascience). Residual undigested antibody was removed on protein G sepharose beads (Pierce). SDS-PAGE revealed no full-length antibody was present in the Fab fragment fraction.

Westerns were probed with rabbit anti-Fz (Bastock and Strutt, 2007), mouse monoclonal anti-Fmi 74 (DSHB, Usui et al., 1999), Actin AC-40 mouse monoclonal antibody (Sigma) and anti-α-Tubulin DM1A (Sigma).

Supplemental references

Bastock, R., and Strutt, D. (2007). The planar polarity pathway promotes coordinated cell migration during *Drosophila* oogenesis. Development *134*, 3055-3064.

Kitamoto, T. (2001). Conditional modification of behavior in Drosophila by targeted expression of a temperature-sensitive shibire allele in defined neurons. J Neurobiol *47*, 81-92.

Lee, Y.S., and Carthew, R.W. (2003). Making a better RNAi vector for *Drosophila*: use of intron spacers. Methods *30*, 322-329.

Marois, E., Mahmoud, A., and Eaton, S. (2006). The endocytic pathway and formation of the Wingless morphogen gradient. Development *133*, 307-317.

Oda, H., Uemura, T., Harada, Y., Iwai, Y., and Takeichi, M. (1994). A *Drosophila* homolog of cadherin associated with Armadillo and essential for embryonic cell-cell adhesion. Dev Biol *165*, 716-726.

Peifer, M., Sweeton, D., Casey, M., and Wieschaus, E. (1994). wingless signal and Zeste-white 3 kinase trigger opposing changes in the intracellular distribution of Armadillo. Development *120*, 369-380.

Shimada, Y., Usui, T., Yanagawa, S., Takeichi, M., and Uemura, T. (2001). Asymmetric co-localisation of Flamingo, a seven-pass transmembrane cadherin, and Dishevelled in planar cell polarisation. Curr Biol *11*, 859-863.

Strutt, D., Johnson, R., Cooper, K., and Bray, S. (2002). Asymmetric localisation of Frizzled and the determination of Notch-dependent cell fate in the *Drosophila* eye. Curr Biol *12*, 813-824.

Strutt, D.I. (2001). Asymmetric localisation of Frizzled and the establishment of cell polarity in the *Drosophila* wing. Mol Cell *7*, 367-375.

Strutt, H., Price, M.A., and Strutt, D. (2006). Planar polarity is positively regulated by casein kinase Iε in *Drosophila*. Curr Biol *16*, 1329-1336.

Strutt, H., and Strutt, D. (2008). Differential stability of Flamingo protein complexes underlies the establishment of planar polarity. Curr Biol *18*, 1555-1564.

Tanaka, T., and Nakamura, A. (2008). The endocytic pathway acts downstream of Oskar in Drosophila germ plasm assembly. Development *135*, 1107-1117.

Tree, D.R.P., Shulman, J.M., Rousset, R., Scott, M.P., Gubb, D., and Axelrod, J.D. (2002). Prickle mediates feedback amplification to generate asymmetric planar cell polarity signalling. Cell *109*, 371-381.

Usui, T., Shima, Y., Shimada, Y., Hirano, S., Burgess, R.W., Schwarz, T.L., Takeichi, M., and Uemura, T. (1999). Flamingo, a seven-pass transmembrane cadherin, regulates planar cell polarity under the control of Frizzled. Cell *98*, 585-595.
