# Supplementary figures and images for: Dynamics of Core Planar Polarity Protein Turnover and Stable Assembly into Discrete Membrane Subdomains
Source: Dev Cell. 2011 Apr 19;20(4):511–25. doi: 10.1016/j.devcel.2011.03.018 (PMC3094756; doi:10.1016/j.devcel.2011.03.018)

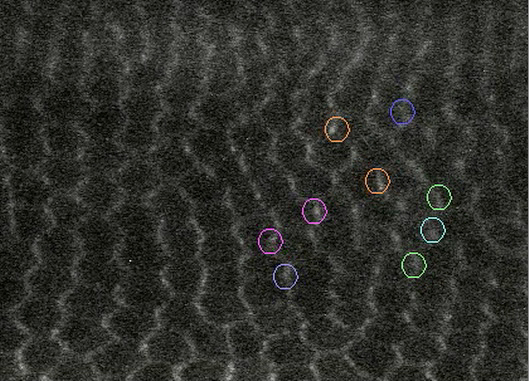

Supplement: Movie S1. Time-Lapse Imaging of Puncta in Pupal Wings — Time-lapse movie of live pupal wing expressing ActP-fz-EYFP at 27-29 hr APF. Images were taken at 5 min intervals for 2 hr. Circles mark individual puncta followed over time; puncta persist in junctions for the duration of the movie. See Figure S3 for stills from this movie. [file mmc2.jpg]
